# Retrieving interpretability to support vector machine regression models in dynamic system identification

**DOI:** 10.3389/frai.2025.1706566

**Published:** 2025-12-19

**Authors:** Johan Pena-Campos, Diego Patino, Carlos Ocampo-Martinez, Julio C. Ramos-Fernández, Margot Salas-Brown, Alexander Caicedo

**Affiliations:** 1 Department of Electronic Engineering, Pontificia Universidad Javeriana, Bogotá, Colombia; 2 Automatic Control Department (ESAII), Universitat Politécnica de Catalunya, Barcelona, Spain; 3 Faculty of Mathematical and Natural Sciences, Universidad Distrital Francisco José de Caldas, Bogotá, Colombia; 4 School of Exact Sciences and Engineering, Universidad Sergio Arboleda, Bogotá, Colombia; 5 Ressolve SAS, Medelln, Colombia

**Keywords:** interpretability, oblique projections, support vector machine, Hammerstein-Wiener models, dynamic systems, system identification

## Abstract

Black-box models, particularly Support Vector Machines (SVM), are widely employed for identifying dynamic systems due to their high predictive accuracy; however, their inherent lack of transparency hinders the understanding of how individual input variables contribute to the system output. Consequently, retrieving interpretability from these complex models has become a critical challenge in the control and identification community. This paper proposes a *post-hoc* functional decomposition algorithm based on Non-linear Oblique Subspace Projections (NObSP). The method decomposes the output of an already identified SVM regression model into a sum of partial (non)linear dynamic contributions associated with each input regressor. By operating in the non-linear feature space, NObSP utilizes oblique projections to mitigate cross-contributions from correlated regressors. Furthermore, an efficient out-of-sample extension is introduced to improve scalability. Numerical simulations performed on benchmark Wiener and Hammerstein structures demonstrate that the proposed method effectively retrieves the underlying partial nonlinear dynamics of each sub-system. Additionally, the computational analysis confirms that the proposed extension reduces the arithmetic complexity from 𝒪(*N*^3^) to 𝒪(*Nd*^2^), where *d* is the number of support vectors. These findings indicate that NObSP is a robust geometric framework for interpreting non-linear dynamic models, offering a scalable solution that successfully decouples blended dynamics without sacrificing the predictive power of the black-box model.

## 1 Introduction

Interpretability of Support Vector Machine (SVM) or Neural Networks (NN) models, examples of black-box models, is a field of study that has recently gained attention, especially for the significant advances of machine learning models and their inclusion in fields such as medicine and law (Barredo Arrieta et al., 2020). For physicians, the accuracy of classification models is as important as understanding why the models provide some results. The lack of understanding of the model performance diminishes the confidence of the specialist in its use, even more so when the model's output differs from the one expected by the specialist. When addressing interpretability of a machine learning model, the aim is to understand how the input parameters influence the model's output. Interpretability can be addressed in two ways: *model* and *instance* explanation approaches. The *local* or *instance* approach tries to explain a prediction for a specific instance, making it valid just for its vicinity. This approach must not be generalized (Burkart and Huber, 2021). In contrast, *global* or *model* interpretability aims to provide information about the model functionality on its whole using only the training data (Barredo Arrieta et al., 2020; Burkart and Huber, 2021).

The framework of interpretability/explainability is still wide and open. In (Luckey et al. 2022), the authors establish an explainable artificial intelligence (XAI) pipeline, where the explanation and interpretation are processes within this pipeline. The goal of the explanation is to identify the most relevant features that influence the classifier decision, i.e., illustrate which input features contribute the most to producing a decision. In the next process, interpretation, the features previously identified are associated with the problem-specific domain, i.e., mapping an abstract concept into a domain that makes sense to humans. Further, in (Barredo Arrieta et al. 2020) the authors define interpretability as a passive characteristic of a model, part of its design, at the level that it has a sense for a human observer; and explicability as the active characteristic that clarify or detail the internal functioning in a model. In (Burkart and Huber 2021), the authors address three concepts: (i) the interpretable models, which are entirely understandable and are built naturally or by using design principles; (ii) the approach of fitting a surrogate model that approximates a black box through local or global interpretable models, and (iii) the process of generating a local or global explanation. In addition, the development of interpretable models can be divided into two groups, according to the moment when interpretability/explainability is applied. If the explanation is produced ante-hoc, they can be called Interpretable (Burkart and Huber, 2021) or Transparent models (Barredo Arrieta et al., 2020; Luckey et al., 2022) whilst explainable models with *post-hoc* explanations.

Linear regression is considered a white box or an interpretable model since it is possible to know how each input variable has contributed to the output. White-box models are characterized for having *ante-hoc* interpretability, which means that they are interpretable on their own (Burkart and Huber, 2021). However, linear regression might lack accuracy in its predictions since it is not able to model nonlinearities that are not explicitly defined in the model design (Harrell et al., 2001). In contrast, black-box models have high accuracy and can adapt to nonlinearities but lack interpretability. For black-box models, *post-hoc* interpretability can be achieved through a global model-agnostic explanator (Barredo Arrieta et al., 2020), which is a white-box surrogate model that simulates the behavior of the black-box model (Burkart and Huber, 2021).

In recent years, powerful *post-hoc* and model-agnostic frameworks have become foundational to XAI. Notably, Local Interpretable Model-agnostic Explanations (LIME) (Ribeiro et al., 2016) and SHapley Additive exPlanations (SHAP) (Lundberg and Lee, 2017) are designed to explain the predictions of any classifier or regressor. These methods are highly effective for feature attribution, providing importance scores that quantify the contribution of each individual feature to a specific prediction.

However, applying these methods directly to dynamic systems is non-trivial, as standard implementations (e.g., KernelSHAP) often assume feature independence, which is fundamentally violated by the temporal correlations (autocorrelation) inherent in time-series data. Although adaptations for time-series have been proposed (Jutte et al., 2025; Sen et al., 2025; Theissler et al., 2022), their objective remains providing saliency scores (e.g., the importance of *u*[*n*−*k*] at a specific time step *k*) rather than retrieving a complete functional dynamic.

Other families of XAI methods face similar limitations in this context. Gradient-based methods, such as Grad-CAM, have been adapted for 1D signals (Selvaraju et al., 2016; Aquino et al., 2022), but they are (i) model-specific to neural networks, requiring access to gradients and internal feature maps, and thus inapplicable to kernel-based models like SVMs, and (ii) focused on identifying saliency (i.e., which parts of the input were most critical), not decomposing the output.

Furthermore, visualization methods like Partial Dependence Plots (PDP) and Individual Conditional Expectation (ICE) plots, which do attempt to show a functional relationship, also struggle with dynamic systems. Their reliance on marginalizing features fails when strong correlations exist—as they always do between lagged regressors (*u*[*n*−1], *u*[*n*−2], etc.). This fact can lead to averaging over “impossible” regions of the feature space, yielding unreliable results (Rojat et al., 2021; Angelini et al., 2023; Shi et al., 2023).

This work, in contrast, addresses a different objective. The goal of the NObSP extension is not feature attribution but functional decomposition. The methodology leverages the specific geometric properties of kernel methods to reconstruct the entire partial (non)linear dynamic contribution of each input regressor (i.e., **ŷ**_*l*_) as an additive component of the total system output. As demonstrated with the Wiener and Hammerstein examples, this decompositional approach, built on oblique projections specifically designed to handle correlated regressors, allows for the retrieval of the underlying sub-system dynamics—a different and more holistic form of interpretation than feature attribution or saliency mapping.

Considering nonlinearities, additive models can decompose the output of a nonlinear regression as the sum of the partial (non)linear contributions of each input variable and their interaction effects. In this context, some approaches already reported in the literature are sparse additive models (Ravikumar et al., 2009), functional ANOVA models (Abramovich and Angelini, 2006), and neural additive models (Agarwal et al., 2021). However, these methods require to specify, a priori, which are the most relevant input variables and interaction effects of interest. This restriction, imposed during the model definition, conditions the functionality of the methods. In contrast, black-box models, such as SVM and NN, do not need to define the variables of interest a priori, but they use all the available input-output observations to find the model that better fits the data. These black-box models generally have greater accuracy in their predictions but lack interpretability (DeVore et al., 2011). Nevertheless, in (Rudin 2019), the authors express that it is a myth that there is necessarily a trade-off between accuracy and interpretability. Additionally, the authors mention that there exists a widespread belief that more complex models (black box) are more accurate, which often is not true, especially when the data are structured with a suitable representation in terms of naturally meaningful features.

SVM is a popular non-parametric framework that uses input data and their targets to estimate a model, which can be employed to generate predictions on unseen data (Vapnik, 1999). SVM produces a *black-box* model that fits the data but does not facilitate the interpretation of the results. As in (Caicedo et al. 2019), in this article, the interpretability is understood as “the property of a model to express the output into additive terms of the partial (non)linear contributions of the input variables.” Interpretability of Least-Squares Support Vector Machine (LS-SVM) has been addressed by employing a truncated multinomial expansion for classifiers (Van Belle and Lisboa, 2014), and using oblique subspace projections for regression models (Caicedo et al., 2019). In (Ravikumar et al. 2009) and (Abramovich and Angelini 2006), the authors propose to retrieve the interpretability of SVM forcing the model to be adjusted from prior knowledge, identifying the contributions of each input variable main effects and interaction effects. Here, the designer needs to define a priori, which input variables and interaction effects of interest are essential for the model. Other approaches use a geometric framework, decomposing the estimated observation vector as a linear additive term through oblique subspace projections (Bring, 1996). This approach is similar to the one proposed by (Caicedo et al. 2019), where they use a nonlinear extension to oblique subspace projections (NObSP). Here, they used a static LS-SVM regression and considered that in the dual space, i.e., the transform space, the underlying model that relates the input and output variables is linear. Therefore, in this dual space the model can be decomposed into additive components. In (Caicedo et al. 2019), they proposed to generate a basis for each subspace of interest using appropriate kernel evaluations, i.e., subspaces that span the (non)linear transformation of each input variable and their interaction effects. They demonstrate the use of NObSP through toy examples and showcase its application in the manufacturing industry using data from the compressive strength dataset from the University of California, Irvine (UCI) machine learning repository (Yeh, 1998). NObSP retrieves the functional relationships between the input and output variables for a static regression model using LS-SVM, even in the presence of correlated inputs. In addition, it does not require for the designer to define a priori the input variables and the interaction effects of interest.

While the previous discussion centered on static regression, the utility of black-box models extends prominently to the identification of nonlinear dynamic systems. In this context, (Gonzalez-Olvera and Tang 2010) have proposed recurrent NN to identify dynamic systems, and (Forgione et al. 2023) proposed a methodology to adapt the identified model using recurrent NN to the changes present in a non-stationary nonlinear system. Moreover, (Yazdani et al. 2020) used deep learning algorithms to estimate the parameters of a differential equation that models a biological system. Likewise, (Candon et al. 2022) compared the performance of multiple-input-single-output (MISO) system identification from linear regression models, Artificial NN, and deep learning strategies in the prediction of the representative bending and torsional load spectra on an aircraft wing based on strain sensors. Besides, (Resendiz-Trejo et al. 2006) used a recursive SVM for MISO nonlinear system online identification, improving computational cost compared to SVM, while (Espinoza et al. 2005) used a partially linear model with an LS-SVM to identify a combined linear-nonlinear model, with fewer parameters, better generalization ability and performance than a full nonlinear black-box model. In addition, Li J. et al. (2022) and (Zong et al. 2021) considered block-oriented system identification approaches, where the nonlinear system is represented as an interconnection of linear and nonlinear blocks. Some examples of these nonlinear systems structures are a Wiener system, i.e., a linear block followed by a nonlinear block, and a Hammerstein system, i.e., a nonlinearity followed by a linear block (Li J. et al., 2022). In this context, some methodologies have been developed for the use of SVM and LS-SVM for the identification of Wiener systems (Castro-Garcia and Suykens, 2016; Bottegal et al., 2018; Bottegai et al., 2017), Hammerstein-Wiener systems (Goethals et al., 2005), and Wiener-Hammerstein systems (Falck et al., 2009). More recent works on these block structures use Particle Swarm Optimization (PSO) to obtain the parameters of the Hammerstein-Wiener nonlinear system, including the time delay (Li J. et al., 2022), and to identify the model in the presence of scarce measurements (Zong et al., 2021). In (Li F. et al., 2022) and (Li et al. 2023c), the authors present a decouple identification scheme model for nonlinear systems through a structure of a Hammerstein system based on a neural fuzzy network and autoregressive exogenous (ARX) model. Finally, this methodology is analyzed with output noise (Li et al., 2023b), and extended for Hammerstein-Wiener (Li et al., 2023a) and Wiener (Li et al., 2024) structures.

Industrial processes like thermal, biological fermentation, chemical processes, pumped-storage power generating systems, and solar-wind hybrid power systems, among others, present nonlinear characteristics. Although the block structure of the Wiener and Hammerstein models is well-known in the literature, their structure helps to reflect the behavior of these types of systems integrating the linear dynamic model with a static memoryless nonlinear model (Li J. et al., 2022; Zong et al., 2021). In addition, time delay phenomenon is also encountered in metallurgy, refining, and glass industries with complex production links, increasing the adjustment time (Li J. et al., 2022).

In the presence of a MISO system, it will be advantageous to fit a general model to the system dynamics and then decompose its output into additive terms, where each term represents the output of a Wiener, Hammerstein, or Wiener-Hammerstein system. Here, a strategy such as NObSP might be of help. However, NObSP has yet to be developed for dynamic systems identification. In addition, to decompose the output of the model using NObSP, it is necessary to compute oblique projection matrices for each input variable, or interaction effect, of interest. This process is computationally expensive with an arithmetic complexity of 𝒪(*N*^3^), where *N* represents the number of observations. Therefore, using NObSP for test data, i.e., out-of-sample extension, is computationally expensive.

Within the context of this study, the primary contributions of this paper are twofold. On one hand, the previous work (Pena-Campos et al., 2023) is extended from static systems to dynamic systems. In this frame, while SVM has been previously employed for system identification purposes, here the methodology adapts the use of NObSP to decompose the output of the identified model of a nonlinear dynamical system into additive components. Where each additive component represents the non-linear dynamic partial contribution of each input variable to the output. These components can be further used in a framework of block system identification to recognize the system components. To the best of the authors' knowledge, the proposed algorithm is the only method capable of retrieving the partial nonlinear dynamic contribution of each independent input without specifying a priori the most relevant input variables or interaction effects of interest and without adjusting the model based on prior domain knowledge. On the other hand, this methodology adapts and validates a computationally more efficient out-of-sample extension, previously introduced by the authors for static regression models (Pena-Campos et al., 2023), for the specific context of nonlinear dynamic system identification. This extension decomposes the output of the model for new testing data using only kernel evaluations and matrix multiplication between the kernel matrix and a set of coefficients. Calculating the matrix multiplication, the extension reduces the arithmetic complexity of the algorithm from 𝒪(*N*^3^) to 𝒪(*Nd*^2^), being *d* the number of support vectors.

This paper is organized as follows: in Section 2, the extension of NObSP for dynamic models using SVM, as well as the out-of-sample extension, are presented. Here, an example is used to evaluate the performance of NObSP. Section 3 presents the results of NObSP using the toy dataset. Section 4 discusses the results and possible improvements to the algorithm. Finally, Section 5 presents some conclusions from the results obtained using the proposed method.

## 2 Methods

The analysis begins by considering the standard mathematical structure used to represent a broad class of discrete-time, multi-input, single-output (MISO) nonlinear dynamic systems (Ljung et al., 2020). This model, often referred to as a Nonlinear Moving Average (NMA) or Nonlinear Finite Impulse Response (NFIR) model, represents the system output as a general, unknown function of a regressor vector composed of present and past inputs.

This generalist structure is foundational in system identification as it can approximate a wide variety of nonlinear dynamics, including, but not limited to, block-oriented structures such as the Wiener and Hammerstein models discussed in this work. The model is defined as:


$$\begin{array}{l} y[n]=\;f(x_{1}[n],\ldots ,x_{1}[n-m_{1}];\ldots ;x_{l}[n],\ldots ,x_{l}[n-m_{l}];\ldots ;\\ \;x_{r}[n],\ldots ,x_{r}[n-m_{r}])+\eta , \end{array}$$ (1)

where *n*∈ℤ^+^ is the discrete-time variable, *y*[*n*]∈ℝ is the *n*^th^ sample of the model's output, and η is a Gaussian noise. The function *f*(·) is an unknown nonlinear continuous map that depends on a regressor vector built from the history of the *r* input variables *x*_*l*_[*n*]∈ℝ. Each of the *r* inputs contributes a block of its current value (e.g., *x*_*l*_[*n*]) and its own *m*_*l*_ past delays (e.g., *x*_*l*_[*n*−1], …, *x*_*l*_[*n*−*m*_*l*_]). Here, *r* represents the number of physical input variables (e.g., voltage, flow rate), and *m*_*l*_ represents the system's memory (number of delays) associated with that specific *l*^th^ input. Therefore, the total dimension of the regressor vector is the sum of all these contributions: *r* (for the *r* current inputs) plus the sum of all individual delays, **M**, where $\text{M}=\overset{r}{\underset{l=1}{\sum }}m_{l}$. The total dimension is thus *r*+**M**. The Gaussian noise term η is included to represent unmodeled dynamics or measurement noise, thus creating a more realistic identification scenario. Now, let consider the decomposition of *y*[*n*] as an additive sum of the form


$$\begin{array}{l} y[n]=\sum_{l=1}^{r}f_{l}\left(x_{l}[n],\ldots ,x_{l}[n-m_{l}]\right)+\\ \;\sum_{l=1}^{r}\sum_{h>l}^{r}f_{l,h}(x_{l}[n],\ldots ,x_{l}[n-m_{l}];\ldots ;x_{h}[n],\ldots ,\\ \;x_{h}[n-m_{h}])+G+\eta , \end{array}$$ (2)

where $f_{l}:{\mathbb{R}}^{m_{l}+1}\to \mathbb{R}$ represents the map that indicates the (non)linear contribution of the *l*^th^-input variable on the output, $f_{l,h}:{\mathbb{R}}^{m_{l}+m_{h}+2}\to \mathbb{R}$ represents the map that indicates the (non)linear second-order interaction contribution of the variables *x*_*l*_ and *x*_*h*_ on the output, and **G**∈ℝ represents the (non)linear contributions of higher order interactions.

The main goal of this paper is to introduce an algorithm that allows the decomposition of the function *f*, which represents the nonlinear dynamic system presented in Equation 1, into the sum presented in Equation 2. In this framework, the term **G** represents the blended contribution of all second- and higher-order interactions. A primary objective of the proposed methodology is to quantify the relative importance of this term. As will be detailed in Section 2.2 (see Equation 13), the magnitude of this residual term—i.e., the difference between the full model output and the sum of the first-order components—is used precisely to determine when higher-order interactions are dominant or when the system can be considered negligibly non-additive.

### 2.1 Support vector machines for system identification

SVM is a kernel-based methodology that can be used either for regression or classification problems. A system identification problem can be formulated as a regression problem, where the input variables contain a temporal window of the input data. In the primal space, the model has the form


$$\hat{y}[n]=\omega^{T}\varphi (x[n])+b,$$ (3)

where ŷ[*n*]∈ℝ is the output of the model, **x**[*n*]∈ℝ^*r*(*m*+1)^ is the vector containing the inputs of the model and their delays, *r* represents the number of input variables, *m* is the length of the temporal window and *b* is the bias term. For simplicity, it is assumed that all variables have the same number of delays in the model, but this is not a restriction. In fact **x**[*n*] is a column vector of the form $\text{x}\left[n\right]={\left[{\text{x}}_{1}\left[n\right],\cdots \;,{\text{x}}_{r}\left[n\right]\right]}^{T}$, with ${\text{x}}_{l}\left[n\right]\in {\mathbb{R}}^{\left(m+1\right)}$ and **x**_*l*_[*n*] = [*x*_*l*_[*n*], ⋯ , *x*_*l*_[*n*−*m*]] is the entry for the *l*^th^-input regressor (e.g., the *u*_1_ block in the example of the previous section) and *T* represents the transpose. For simplicity, this study assumes a uniform memory *m* for all inputs. The selection of an optimal *m* (or different *m*_*l*_ for each input) is a separate model selection problem, which falls outside the scope of interpreting the already-identified model. Besides, φ:ℝ^*r*·(*m*+1)^ → ℝ^*p*^ represents the nonlinear mapping of the input vector into a high-dimensional (and potentially infinite) feature space. This “kernel trick" is the core of the SVM, allowing it to solve a linear regression problem in the feature space, which corresponds to a powerful nonlinear regression in the original input space (Suykens J. A. K. et al., 2002). Moreover, **ω**∈ℝ^*p*^ are the weights of the SVM model. Depending on the selection of the kernel function, the dimension *p* of the nonlinear mapping can be infinite.

Before the decomposition of Equation 2 can be performed, an accurate, non-parametric model of the full function *f* in Equation 1 must first be identified. Therefore, the process begins by determining how to obtain *f* using SVM.

To make this structure concrete, consider a simple MISO system with *r* = 2 inputs (*u*_1_, *u*_2_) and a uniform memory of *m* = 2. The general input vector **x**[*n*] from Equation 3 for any time *n* is constructed by “stacking" the present and past values of all inputs $\text{x}\left[n\right]=\left[{\left[u_{1}\left[n\right],u_{1}\left[n-1\right],u_{1}\left[n-2\right]\right]}^{T}[{\left[u_{2}\left[n\right],u_{2}\left[n-1\right],u_{2}\left[n-2\right]\right]}^{T}\right]$.

The SVM model will learn a single function *f*(**x**[*n*]) based on this vector. The goal of NObSP is to decompose the output of this function, ŷ[*n*], back into two components: one attributable to the block ${\left[u_{1}\left[n\right],u_{1}\left[n-1\right],u_{1}\left[n-2\right]\right]}^{T}$ and another attributable to the block ${\left[u_{2}\left[n\right],u_{2}\left[n-1\right],u_{2}\left[n-2\right]\right]}^{T}$.

Given the training set ${\left\{{\text{x}}^{\left(i\right)},y^{\left(i\right)}\right\}}_{i=1}^{N}$, where the superindex (*i*) indicates the *i*^th^ observation, the SVM regression problem can be formulated as follows (Suykens J. A. K. et al., 2002):


$$\begin{array}{l} \;\underset{\omega ,b,\xi ,\xi^{*}}{\text{min}}\;\frac{1}{2}\omega^{T}\omega +c\sum_{i=1}^{N}\left(\xi^{\left(i\right)}+\xi^{*(i)}\right)\\ \text{subject to }y^{\left(i\right)}-\omega^{T}\varphi (x^{\left(i\right)})-b\leq \epsilon +\xi^{\left(i\right)},\\ \;\omega^{T}\varphi (x^{\left(i\right)})+b-y^{\left(i\right)}\leq \epsilon +\xi^{*(i)},\xi^{\left(i\right)},\\ \;\xi^{*(i)}\geq 0,\forall i=1,\ldots ,N \end{array}$$ (4)

where *c* is the regularization constrain parameter, in the Vapnik ϵ-insensitive loss function ϵ is the tolerated error for the regression model, ξ^(*i*)^ and ξ^*(*i*)^ are slack variables that manage data outside the ϵ-sensitive tube, and *N* is the number of observations. Taking the Lagrangian and solving for the Karush-Kuhn-Tucker conditions for optimally, the solution to problem Equation 4 in matrix form is given by Suykens J. A. K. et al. (2002).


$$\begin{array}{l} ŷ=\text{Ω}\alpha +b, \end{array}$$ (5)

where **ŷ**∈ℝ^*N*^ is a column vector representing the output of the model, with components such that $ŷ={\left[{\hat{y}}^{\left(1\right)},\cdots \;,{\hat{y}}^{\left(N\right)}\right]}^{T}$, **Ω**∈ℝ^*N*×*d*^, with *d* the number of support vectors, **Ω**^(*i, j*)^  =  φ(^**x**^(*i*)^)*T*^φ(**x**^(*j*)^)  =  K(**x**^(*i*)^, **x**^(*j*)^) is the *ij*^th^ element of the kernel matrix, and K(·, ·) is the kernel function, considering *x*^(*i*)^ and *x*^(*j*)^ the *i*-th and *j*-th observation, respectively. Besides, **α**∈ℝ^*d*^ is a column vector containing the Lagrange multipliers and *b* is the bias term. To solve the problem (Equation 4), it is necessary to find the values for the kernel hyper-parameters, as well as the model order *m*, that minimizes the cost function.

Furthermore, for NObSP, it is crucial to normalize the input data and center the kernel matrix. This normalization can be done by subtracting the bias term in the estimated output such that (Suykens J. et al., 2002).


$$\begin{array}{l} ŷ={\text{Ω}}_{C}\alpha , \end{array}$$ (6)

where **Ω**_*C*_ = **M**_1_**ΩM**_2_ is the centered kernel matrix, ${\text{M}}_{1}={\text{I}}_{N}-1_{N}1_{N}^{T}/N$ and ${\text{M}}_{2}={\text{I}}_{d}-1_{d}1_{d}^{T}/d$ are centering matrices, with **I**_*N*_ the identity matrix of N size, and $1_{N}\in {\mathbb{R}}^{N}$ is a column vector of unitary entries. The *ij*^th^ entry of the centered kernel matrix is given by ${\text{Ω}}_{C}^{\left(i,j\right)}={\left(\varphi \left({\text{x}}^{\left(i\right)}\right)-\mu_{\varphi }\right)}^{T}\left(\varphi \left({\text{x}}^{\left(j\right)}\right)-\mu_{\varphi }\right)$, with $\mu_{\varphi }\in {\mathbb{R}}^{p}$ the mean value of the nonlinear transformation of the input variables.

Since SVM is used to identify the nonlinear model, then the SVM model is able to represent, in a non-parametric way (Equation 1). Then, the function *f*(·) should be a function that lies in a Hilbert space, i.e., *f*(·) is a smooth and continuous function.

It is critical to understand the relationship between the SVM model (Equation 3) and the decomposition goal (Equation 2). The SVM does not inherently support the decomposition. Rather, the trained SVM model becomes the black-box function *f* that is to be interpreted. The SVM provides a non-parametric representation of the overall system dynamics, ŷ[*n*] = *f*(**x**[*n*]).

The NObSP methodology, introduced next, is the *post-hoc* tool that operates on this already-trained SVM model. NObSP takes the full model *f* and applies a geometric decomposition to retrieve the non-parametric partial contributions *f*_*l*_(·), *f*_*l, h*_(·), etc., which aligns with the additive structure defined in Equation 2.

### 2.2 Nonlinear oblique subspace projections for SVM

The core challenge in functional decomposition, and the primary motivation for NObSP, arises when input regressors are correlated. This collinearity in the input space is the underlying cause for the subspaces in the nonlinear feature space (the high-dimensional space mapped by φ(·)) to be non-orthogonal. In this feature space, the SVM model itself is linear (Equation 5). However, the input correlation means that their respective feature subspaces are not orthogonal; their intersection is not null and they are overlapping.

A standard orthogonal projection (which assumes orthogonality) would incorrectly capture energy from all other overlapping subspaces, leading to an erroneous decomposition. This is precisely the problem Nonlinear Oblique Subspace Projections (NObSP) is designed to address. NObSP operates geometrically in this feature space, using oblique projections to mitigate the cross-contributions from one overlapping subspace to another and isolate only the unique contribution of each regressor (Caicedo et al., 2019; Bring, 1996). It is important to note, however, that in the presence of extremely high correlations, the method can become numerically unstable and may produce unreliable results.

**Figure 1:**
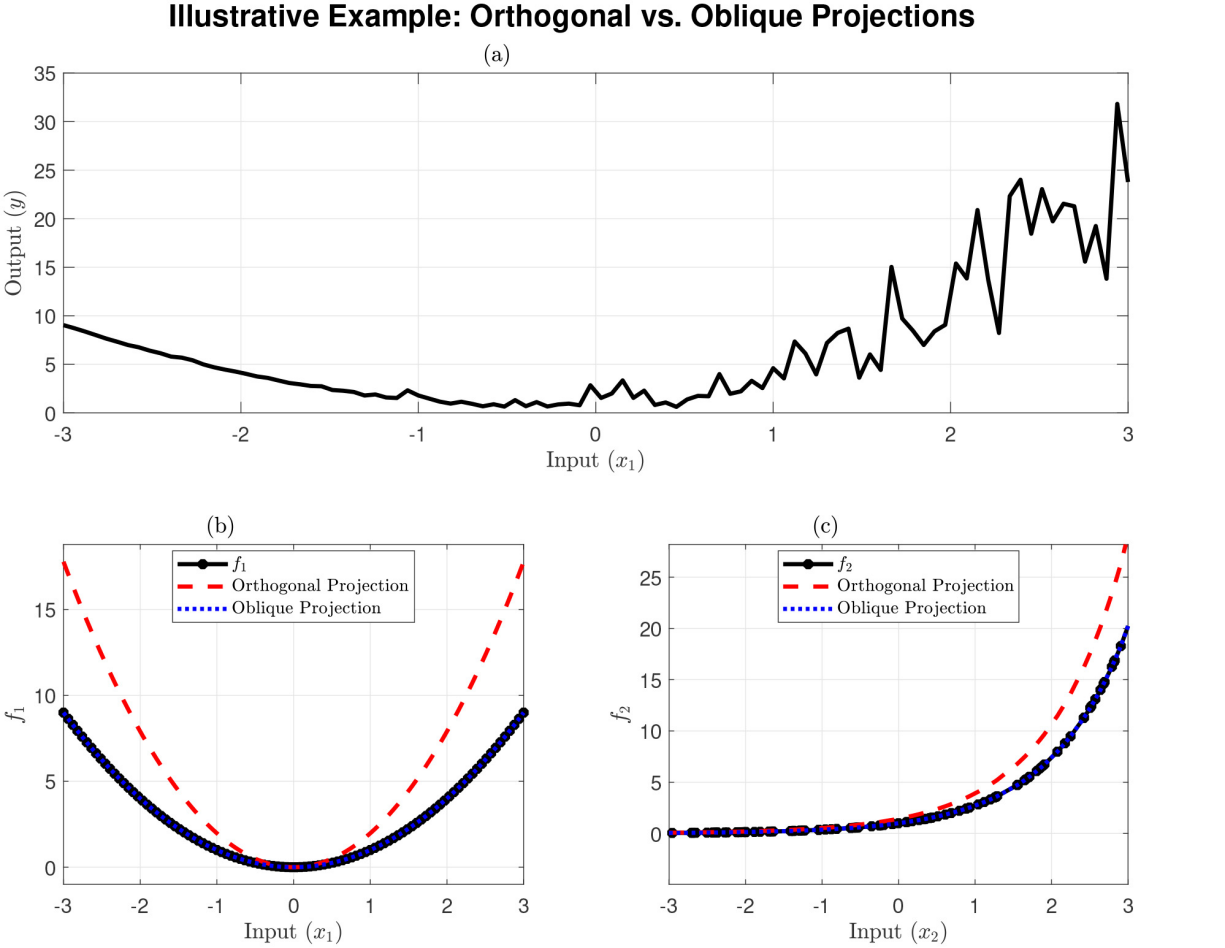
Illustrative example of NObSP for correlated regressors. **(a)** Shows the total observed signal *y*. **(b)** Shows the retrieval of $f_{1}\left(x_{1}\right)=x_{1}^{2}$. **(c)** Shows the retrieval of *f*_2_(*x*_2_) = exp(*x*_2_). In both **(b, c)**, the orthogonal projection (red dashed line) fails to match the ground truth (black line), while the oblique projection (NObSP, blue dotted line) successfully retrieves the true component.

To illustrate this principle visually, a simple simulation was performed. Consider a MISO system *y* = *f*_1_(*x*_1_)+*f*_2_(*x*_2_), where the true components are $f_{1}\left(x_{1}\right)=x_{1}^{2}$ and *f*_2_(*x*_2_) = exp(*x*_2_). The inputs were generated such that *x*_2_ is a noisy, correlated version of *x*_1_ (e.g., *x*_2_≈*x*_1_+*c*). The results are shown in Figure 1. It is important to note that while the data was generated with a dependency, the NObSP method treats *x*_1_ and *x*_2_ as independent regressors. When the algorithm isolates the *f*_1_ contribution (conceptually “zeroing” *x*_2_), it is treating *x*_2_ as an independent subspace, not as a constant *c*.

As predicted by the theory, Figures 1b, c show that the orthogonal projection fails, retrieving a blended, incorrect signal. In contrast, the NObSP (oblique) projection successfully isolates the unique contribution of both *f*_1_(*x*_1_) and *f*_2_(*x*_2_), matching the ground truth. This simple example demonstrates the principle that NObSP extends to the high-dimensional feature space of the SVM. The following defines this nonlinear extension formally.

Let define **A** = [**A**_*l*_ **A**_(*l*)_] as a matrix where its columns span the subspace 𝒱⊂ℝ^*N*^, with **A**∈ℝ^*N*×*r*^, ${\text{A}}_{l}\in {\mathbb{R}}^{N\times q}$ a partition of **A** that spans the subspace 𝒱_*l*_⊂𝒱 and ${\text{A}}_{\left(l\right)}\in {\mathbb{R}}^{N\times \left(r-q\right)}$ a partition of **A** that spans the subspace 𝒱_(*l*)_⊂𝒱. Considering 𝒱 = 𝒱_1_⊕⋯⊕𝒱_*l*_⊕⋯⊕𝒱_*r*_, with ⊕ the direct sum operator and *r* the number of regressor subspaces embedded in **A**, then the matrix that represents the oblique subspace projection onto 𝒱_*l*_ along 𝒱_(*l*)_ = 𝒱_1_⊕⋯⊕𝒱_*l*−1_⊕𝒱_*l*+1_⊕⋯⊕𝒱_*r*_, i.e., the matrix that projects onto the subspace of the input regressor **x**_*l*_ along the complementary regressors subspace **x**_(*l*)_, is denoted by **P**_*l*/(*l*)_ and it is defined as


$$\begin{array}{l} {\text{P}}_{l/\left(l\right)}={\text{A}}_{l}{\left({\text{A}}_{l}^{\text{T}}{\text{Q}}_{\left(l\right)}{\text{A}}_{l}\right)}^{†}{\text{A}}_{l}^{\text{T}}{\text{Q}}_{\left(l\right)}, \end{array}$$ (7)

where † is the generalized inverse of Moore-Penrose (pseudoinverse), **Q**_(*l*)_ is the orthogonal projector onto $\text{Null}\left({\text{A}}_{\left(l\right)}^{\text{T}}\right)\subset {𝒱}_{\left(l\right)}^{⊥}$, computed as **Q**_(*l*)_ = **I**_*N*_−**P**_(*l*)_, **P**_(*l*)_ is the orthogonal projector needed to find the base onto 𝒱_(*l*)_, and computed as ${\text{P}}_{\left(l\right)}={\text{A}}_{\left(l\right)}{\left({\text{A}}_{\left(l\right)}^{\text{T}}{\text{A}}_{\left(l\right)}\right)}^{†}{\text{A}}_{\left(l\right)}^{\text{T}}$, being Null(·) the null space of a matrix.

Considering ŷ_*l*_ = *f*_*l*_(**x**_*l*_), as presented in Equation 2, the objective is to find an oblique projection matrix such that


$$\begin{array}{l} {ŷ}_{l}={\text{P}}_{l/\left(l\right)}ŷ, \end{array}$$ (8)

where **ŷ** is the output of the SVM regression model and **P**_*l*/(*l*)_ is the oblique projection matrix onto the subspace spanned by the nonlinear transformation of the *l*^th^ input variable, along the direction defined by the other variables, represented by (*l*). As presented in (Caicedo et al. 2019), proper kernel evaluations can be used to obtain these projection matrices. Therefore, a base for the subspace that represents the nonlinear transformation of the input regressor **x**_*l*_ can be found by using the kernel matrix **Ω**_*l*_, where


$$\begin{array}{l} {\text{Ω}}_{l}^{\left(i,j\right)}=\text{K}\left({\text{x}}_{l}^{\left(i\right)},{\text{x}}^{\left(j\right)}\right), \end{array}$$ (9)

with ${\text{x}}_{l}^{\left(i\right)}=\left[0,\cdots \;,x_{l}\left[i\right],\cdots \;,x_{l}\left[i-m\right],\cdots \;,0\right]$. In the same way, a basis for the subspace that represents the nonlinear transformation of the complementary regressors **x**_(*l*)_ is defined by the kernel matrix **Ω**_(*l*)_, where


$$\begin{array}{l} {\text{Ω}}_{\left(l\right)}^{\left(i,j\right)}=\text{K}\left({\text{x}}_{\left(l\right)}^{\left(i\right)},{\text{x}}^{\left(j\right)}\right), \end{array}$$ (10)

and ${\text{x}}_{\left(l\right)}^{\left(i\right)}=\left[x_{1}\left[i\right],\cdots \;,x_{1}\left[i-m\right],\cdots \;,x_{l-1}\left[i\right],\cdots \;,x_{l-1}\left[i-m\right],0,\cdots \;,0,x_{l+1}\left[i\right],\cdots \;,x_{l+1}\left[i-m\right],\cdots \;,x_{r}\left[i\right],\cdots \;,x_{r}\left[i-m\right]\right]$. In summary, the column space of **Ω**_*l*_ represents the subspace for the nonlinear transformation of the variable **x**_*l*_, onto which it will project the estimated output of the model, and the column space of **Ω**_(*l*)_ represents the reference subspace for the projection. Using both kernel matrices, the nonlinear oblique projection can be defined as


$$\begin{array}{l} {\text{P}}_{l/\left(l\right)}={\text{Ω}}_{l}{\left({\text{Ω}}_{l}^{\text{T}}{\text{Q}}_{\left(l\right)}{\text{Ω}}_{l}\right)}^{†}{\text{Ω}}_{l}^{\text{T}}{\text{Q}}_{\left(l\right)}, \end{array}$$ (11)

where **Q**_(*l*)_ = **I**_*N*_−**P**_(*l*)_, and ${\text{P}}_{\left(l\right)}={\text{Ω}}_{\left(l\right)}{\left({\text{Ω}}_{\left(l\right)}^{\text{T}}{\text{Ω}}_{\left(l\right)}\right)}^{†}{\text{Ω}}_{\left(l\right)}^{\text{T}}$. For more details on the proof of Equation 11, please refer to (Caicedo et al. 2019).

The nonlinear version for the oblique projections presented in Equation 11 can be used to decompose the output of a dynamic model into additive components, each representing the nonlinear dynamic contribution of the input variables on the output. In this study, the focus is placed on retrieving the *first-order main effects* (i.e., **ŷ**_*l*_), as this main effect provides the most direct interpretation of each input regressor's partial contribution. It is crucial to discuss the role of second- and higher-order terms in this framework.

While the NObSP methodology can be formally extended to compute interaction effects (e.g., **ŷ**_*l, h*_) by defining a target subspace **Ω**_*l, h*_ (Caicedo et al., 2019), this interaction effect introduces a significant interpretive challenge. The fundamental issue lies not in the projection method, but in the functional nature of the (unknown) interaction itself.

NObSP, by design, finds the total partial contribution of a regressor (e.g., **ŷ**_*l*_). If the underlying model contains a simple multiplicative interaction (e.g., *f*_*l, h*_ = *x*_1_·*x*_2_), the projection—which is analogous to setting other inputs to zero—successfully isolates the main effects, as the interaction term **x**_1_·0, vanishes.

However, this behavior cannot be guaranteed for an arbitrary nonlinear function. Consider, for example, a model with a non-separable additive interaction, such as *f*(*x*_1_, *x*_2_) = *f*_*l*_(*x*_1_)+*f*_*h*_(*x*_2_)+cos(*x*_1_+*x*_2_). In this case, the NObSP projection for *x*_1_ (analogous to setting *x*_2_ = 0) will correctly retrieve the total contribution **ŷ**_*l*_≈*f*_*l*_(*x*_1_)+cos(*x*_1_), and the projection for *x*_2_ will retrieve **ŷ**_*h*_≈*f*_*h*_(*x*_2_)+cos(*x*_2_). The interaction term cos(·) becomes additively coupled (or blended) with both main effects.

Thus, NObSP is performing correctly; it reveals exactly what the black-box model is doing. The challenge, which remains an open research problem, is one of decoupling: how to further separate the “pure” main effect (e.g., *f*_*l*_(*x*_1_)) from its share of the non-separable interaction (e.g., cos(*x*_1_)) when a pre-defined structure cannot be imposed.

Given this challenge, this paper adopts a pragmatic and clear quantitative approach. First, the sum of all identified main effects is computed as


$$\begin{array}{l} {ŷ}_{\text{main}}=\overset{r}{\underset{l=1}{\sum }}{ŷ}_{l}. \end{array}$$ (12)

Then, the interaction residual, **r**_interaction_, is defined as the difference between the full black-box model output and the sum of the main effects, i.e.,


$$\begin{array}{l} {\text{r}}_{\text{interaction}}=ŷ-{ŷ}_{\text{main}}=ŷ-\overset{r}{\underset{l=1}{\sum }}{ŷ}_{l}. \end{array}$$ (13)

This residual **r**_interaction_ serves as a direct, quantitative measure of the *total contribution of all second- and higher-order non-separable interactions*. The magnitude of this residual (e.g., its variance or RMSE) directly illustrates the relative importance of these higher-order contributions. A small residual indicates the first-order decomposition is a faithful representation (the system is largely additive), while a large residual indicates that complex, high-order interactions are dominant.

Additionally, to apply NObSP to new testing data, the projection matrices should be computed, which imply to find the kernel matrices **Ω**_*l*_ and **Ω**_(*l*)_. These operations are computationally expensive.

### 2.3 Out-of-sample extension for NObSP

The vectors **ŷ**_*l*_ can be seen as the nonlinear contributions on the output of the *l*-th input feature. NObSP provides a way to compute each one of the contributions for a single input instance. To better illustrate this fact, notice that oblique projections arise naturally from a weighted least-squares problem of the form


$$\begin{array}{l} {\hat{\text{α}}}_{l}=\underset{{\text{α}}_{l}}{\min}||{\text{Q}}_{\left(l\right)}\left(\text{y}-{\text{Ω}}_{l}{\text{α}}_{l}\right)|{|}^{2}, \end{array}$$ (14)

with ${ŷ}_{l}={\text{Ω}}_{l}{\hat{\alpha }}_{l}$. Then, given an input instance **x**^(*i*)^∈ℝ^*r*^ the model predictions could be decomposed ${ŷ}^{\left(i\right)}=\overset{r}{\underset{l=1}{\sum }}{ŷ}_{l}^{\left(i\right)}$, where ${ŷ}_{l}^{\left(i\right)}={\text{Ω}}_{l}\alpha_{l}$, ${\text{Ω}}_{l}^{\left(i,j\right)}=\text{K}\left({\text{x}}_{l}^{\left(i\right)},{\text{x}}^{\left(j\right)}\right)$, and ${\text{x}}_{l}^{\left(i\right)}=\left[0,\cdots \;,x_{l}\left[i\right],\cdots \;,x_{l}\left[i-m\right],\cdots \;,0\right]$, where


$$\begin{array}{l} \overset{r}{\underset{k=1}{⋃}}𝒞\left({\text{Ω}}_{x_{k}}\right)\subseteq 𝒞\left(\text{Ω}\right), \end{array}$$

where 𝒞 represents the column space. The values ${ŷ}_{l}^{\left(i\right)}$ indicate the nonlinear contribution of the *l*-th input feature. Therefore,


$$\begin{array}{l} {\hat{\text{α}}}_{l}={\left({\text{Ω}}_{l}^{\text{T}}{\text{Ω}}_{l}\right)}^{†}{\text{Ω}}_{l}^{\text{T}}{\text{y}}_{l}. \end{array}$$ (15)

Finally, considering a new input sample **x** = [*x*_1_[*n*], ⋯ , *x*_1_[*n*−*m*], ⋯ , *x*_*r*_[*n*], ⋯ , *x*_*r*_[*n*−*m*]], the output of the model can be approximated by


$$\begin{array}{l} ŷ\left(\text{x}\right)\approx \overset{r}{\underset{l=1}{\sum }}\text{K}\left({\text{x}}_{l},{\text{x}}_{\text{SV}}\right){\text{α}}_{l}. \end{array}$$ (16)

In this way, for new data points **x**, the output of the model is given by **ŷ**_*l*_ = K(**x**_*l*_, **x**_SV_)**α**_*l*_, where **x**_SV_ are the support vectors and **x**_*l*_ = [0, ⋯ , *x*_*l*_[*n*], ⋯ , *x*_*l*_[*n*−*m*], ⋯ , 0], with *n* representing the time. In addition, the partial nonlinear contribution of the *l*^th^ regressor does not require the computation of the oblique projections.

Algorithm 1 summarizes the computation for the decomposition of the output using NObSP and for the computation of the out-of-sample extension.

Algorithm 1 Nonlinear Subspace Projection (NObSP). 
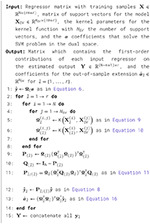

### 2.4 Simulation study

**Figure 2:**
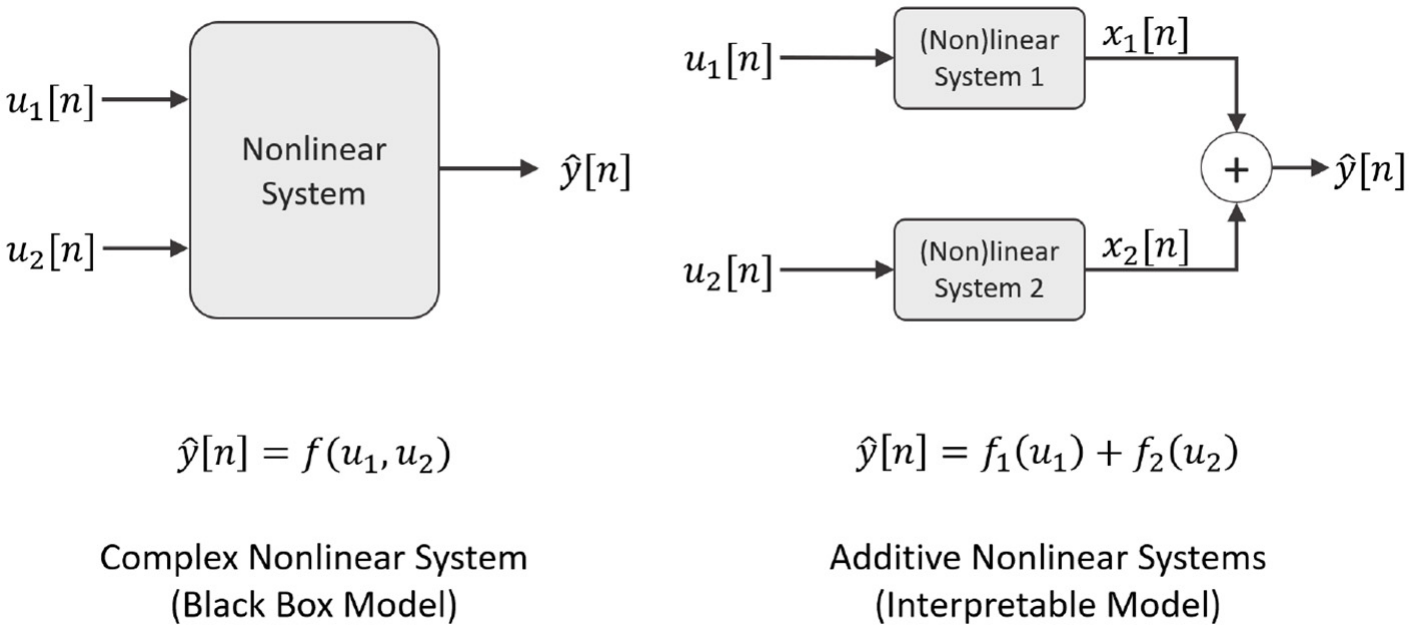
Proposed decomposition scheme using NObSP. The complex black-box model is decomposed into nonlinear systems that represent the decoupled nonlinear contribution of each input variable on the output.

Figure 2 graphically represents the main objective for the simulations presented in this paper. First, an SVM regression model is trained by using the set of observations ${\left\{u_{1}\left[n\right],u_{2}\left[n\right],y\left[n\right]\right\}}_{n=1}^{N}$, where *N* represents the number of samples. Then, the dynamical system is decomposed using NObSP, which produces an additive model. Each component of the decomposed model represents the nonlinear contribution of each input variable, or the interaction effects, on the output. Finding the dynamic functional relation between each input and the output allows understanding the contribution of each variable to the output. The knowledge of the input/output dynamics relation might facilitate the management of each branch and would reduce the complexity of the model or even design simpler and appropriate control systems. For the simulations presented in this paper, interaction effects among the input variables on the output are not considered.

The simulations consider Wiener and Hammerstein block-oriented design for the nonlinear system. Specifically, this paper uses the same system design presented by (Castro-Garcia and Suykens 2016). In (Castro-Garcia and Suykens 2016), the researchers used the *q*-notation for system identification, where *q*^−1^*u*[*n*] = *u*[*n*−1]. For the first branch, as presented by (Castro-Garcia and Suykens 2016), the nonlinear block is given by


$$\begin{array}{l} f_{1}\left[n\right]=z^{3}\left[n\right], \end{array}$$ (17)

where *z*[*n*] is the input sequence to the nonlinear block and *G*_1_(*q*) is a linear discrete-time transfer function defined in the *q*-operator notation:


$$G_{1}\left(q\right)=\frac{B_{1}\left(q\right)}{A_{1}\left(q\right)},$$

where


$$\begin{array}{l} \begin{array}{cc} B_{1}\left(q\right) & =0.0089q^{3}-0.0045q^{2}-0.0045q+0.0089,\\ A_{1}\left(q\right) & =q^{3}-2.5641q^{2}+2.2185q-0.6456. \end{array} \end{array}$$

For the second branch, the nonlinear block is given by


$$\begin{array}{l} f_{2}\left[n\right]=sinc\left(z\left[n\right]\right)z^{2}\left[n\right], \end{array}$$ (18)

where


$$\begin{array}{l} sinc\left(z\left[n\right]\right)=\frac{\sin\left(z\left[n\right]\right)}{z\left[n\right]}, \end{array}$$

and the linear block is given by


$$G_{2}\left(q\right)=\frac{B_{2}\left(q\right)}{A_{2}\left(q\right)},$$

where


$$\begin{array}{l} \begin{array}{cc} B_{2}\left(q\right) & =0.0047q^{3}+0.0142q^{2}+0.0142q+0.0047,\\ A_{2}\left(q\right) & =q^{3}-2.458q^{2}+2.262q-0.7654. \end{array} \end{array}$$

The output of the model is the result of two additive components, one per branch, such that


$$y\left[n\right]=x_{1}\left[n\right]+x_{2}\left[n\right]+\eta ,$$

where η is a Gaussian noise, *x*_1_[*n*] and *x*_2_[*n*] are defined below for each block-structure. The output for the Wiener system is given by


$$y_{W}\left[n\right]=G_{1}\left(q\right)f_{1}\left(u_{1}\left[n\right]\right)+G_{2}\left(q\right)f_{2}\left(u_{2}\left[n\right]\right)+\eta ,$$

where *x*_*i*_[*n*] = *G*_*i*_(*q*)*f*_*i*_(*u*_*i*_[*n*]) represents the linear transformation *G*_*i*_(*q*) applied on the sequence *f*_*i*_(*u*_*i*_[*n*]), while *f*_1_ and *f*_2_ are defined in Equations 17, 18, respectively. Here, *x*_*i*_[*n*] represents the unobservable internal output of the *i*-th branch, which is the “ground truth” signal that the NObSP decomposition aims to retrieve. In addition, the output for a Hammerstein system is given by


$$y_{H}\left[n\right]=f_{1}\left(G_{1}\left(q\right)u_{1}\left[n\right]\right)+f_{2}\left(G_{2}\left(q\right)u_{2}\left[n\right]\right)+\eta ,$$

where *x*_*i*_[*n*] = *f*_*i*_(*G*_*i*_(*q*)*u*_*i*_[*n*]), for *i* = {1, 2}.

Two input signals were created for both scenarios to construct the training and test datasets. For *u*_1_[*n*], *N* samples were drawn from a pseudo-binary random sequence (PBRS). This signal was chosen because its broadband spectral properties are ideal for exciting a wide range of system dynamics, a standard practice in system identification. The signal was generated with the function pbrs, where the parameters used were an order of 99, a length of *N* samples, and a seed of 99 different binary elements, computed using a random sequence obtained from the function rand, both functions from MATLAB R2022b. For *u*_2_[*n*], *N* samples were drawn from a sinusoidal signal $u_{2}\left[n\right]=\sin\left(21\pi \frac{n}{N}\right)$. This signal was chosen to test the model's ability to identify and separate a purely frequency-specific component.

Since the quality of the projections depends on the performance of the model. Several simulations were performed to test the robustness of the projections.^1^

The tests performed are described below. For each simulation test (i.e., each combination of *N*, *m*, SNR, or amplitude ratio), the experiment was repeated 15 times with different random seeds for the noise generation (η). The RMSE values presented in the figures represent the average result of these 15 trials, providing a robust measure of performance.

These simulation parameters were chosen to connect with practical, real-world contexts. The number of samples *N* reflects the data availability from an experiment. The model order *m* represents the system's memory or complexity; a real-world chemical process might have a large *m*, while a simple electronic circuit might have a small *m*. Finally, the SNR reflects the quality of the measurement sensors and the level of ambient noise in a physical plant.

#### 2.4.1 Model order and number of training samples

The impact of the model order had been evaluated *m*, and the number of training samples used to fit the model, *N*. For each pair of variable values, the Root Mean Squared Error (RMSE) was computed between the predicted output and the real output. The RMSE was also calculated for the model output and the estimated projections, i.e., the estimated nonlinear contributions of each branch ${\hat{x}}_{1}\left[n\right]$, and ${\hat{x}}_{2}\left[n\right]$.

For the simulation, the following ranges of values were used: *N* takes values between 50–8,000 observations, while the order of the model, *m*, varies between 5 and 200. Since the Wiener and the Hammerstein structures impose different dynamics on the output signal, such dynamics are expected to affect the optimal values for *N* and *m* in both block-system structures, thereby affecting the projections. The goodness of the fit was evaluated based on the estimations on a test dataset.

#### 2.4.2 Influence of external noise

The robustness of the projections to external noise was evaluated by changing the signal-to-noise ratio (SNR) of the output signal. Simulations had been performed for values of SNR ranging from 0.8 dB up to 18 dB. This simulation used the values for *N* and *m*, in the SVM model, that produced the lowest RMSE.

The impact of changing the amplitude of noise in the output was computed for the model output, as well as for the estimated projections and the out-of-sample extension model (Equation 15).

#### 2.4.3 Influence on the relative difference in amplitude for both branches

As shown in Figure 2, the main objective of NObSP is to decomposed the output **ŷ** into an additive model where **ŷ** = **x**_1_+**x**_2_+**x**_1, 2_. However, it is important to estimate the effect of changing the relative magnitudes of the components, i.e., what happens when the magnitude of **x**_1_ is larger than the magnitude of **x**_2_ and vice versa. In (Caicedo et al. 2019), it was shown that, for the static case, NObSP is able to retrieve the dynamics of different components, even if there were differences in magnitude. The static case algorithm also determined when the contribution to the output of an input regressor was close to zero.

The impact of the changes in relative amplitude between the signal **x**_1_ and **x**_2_ is evaluated for both nonlinear block structures. The relative gain values range from 0.1 to 1, i.e., the amplitude of the signal **x**_1_ varies between 0.1 to 1 times the magnitude of **x**_2_. In total, four simulations had been performed, two for each system structure, one varying the relative amplitude of **x**_1_ using as reference **x**_2_, and varying the relative amplitude of **x**_2_ using **x**_1_ as reference.

## 3 Results

**Figure 3:**
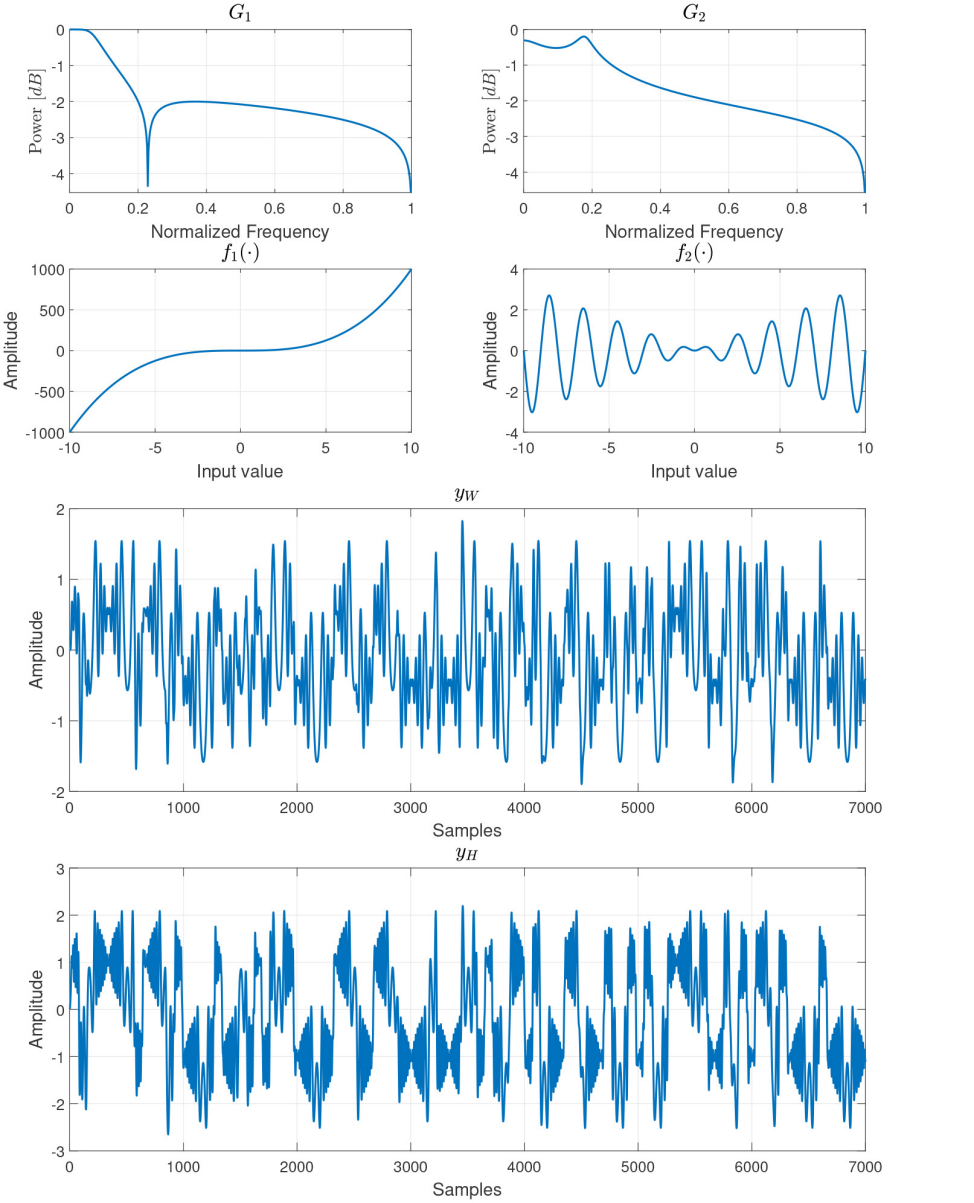
Block system approach. This figure presents the frequency response of the filters, first row, the nonlinear blocks, second row, and the output for the Wiener and the Hammerstein block-system structure, using the PBRS and the sinusoidal signal as input.

In Figure 3, in the top row, it is shown the normalized frequency responses for both linear systems *G*_1_ and *G*_2_. The second row presents the nonlinear functions used for each model branch. On the left, the function $f_{1}\left(n\right)=z^{3}\left[n\right]$ is presented, and on the right, the function $f_{2}\left(n\right)=sinc\left(z\left[n\right]\right)z^{2}\left[n\right]$. The third row indicates the output of the Wiener system, *y*_*w*_[*n*], when using the PBRS signal as input for the first branch and the sinusoid as input for the second branch. The fourth row displays the output for the Hammerstein system, *y*_*H*_[*n*], using the same input configuration as for the Wiener case. As can be seen, the dynamics for both systems are entirely different: *y*_*W*_[*n*] presents a *smoother* behavior, mainly caused by the application of a low-pass filter after the input signals have been non-linearly transformed. While in *y*_*H*_[*n*], the nonlinear function was applied after filtering the input signals, which results in a more complex behavior than *y*_*W*_[*n*].

**Figure 4:**
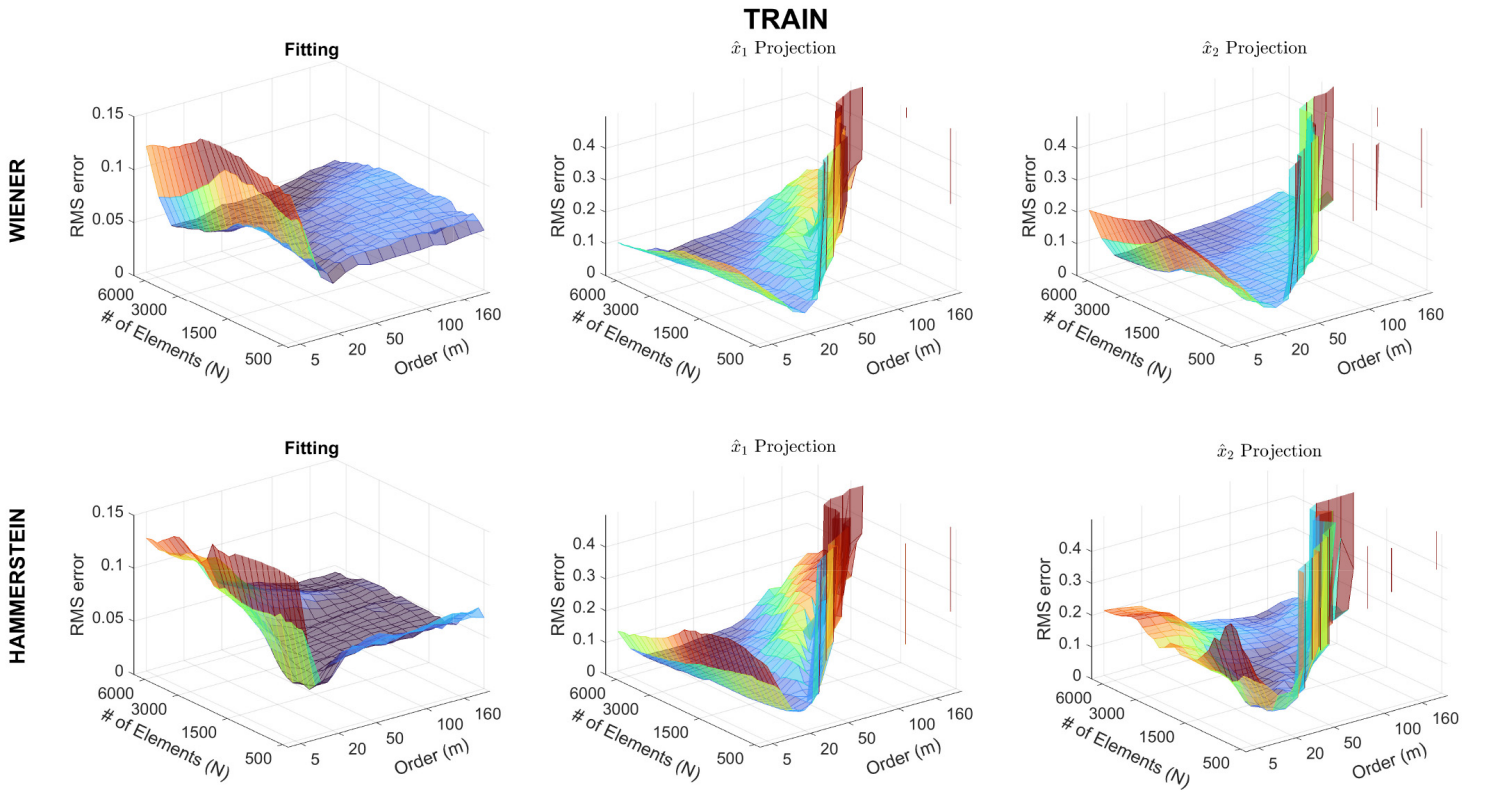
Normalized RMS Error obtained for the fit of the output, and the estimated partial (non)linear contributions of the input regressors on the output.

Figure 4 presents the evolution of the error for the different values of *N* and *m*. The first column shows the RMSE for the global fit of the model, i.e., how the model was able to fit the output using the input signals. The second and the third columns show the RMSE for the estimated projections ${\hat{x}}_{1}$ and ${\hat{x}}_{2}$. The first row indicates the results for the Wiener structure, while the second row displays the results for the Hammerstein structure. Remarkably, for the output, first column, it can be seen that the Wiener and the Hammerstein systems present a different behavior, having a minimum for the RMSE error in different regions of the figure. For the Wiener block structure, the minimum RMSE is given by a low model order, *m*, but a large number of observations *N*. In contrast, the Hammerstein structure also requires a low model order, *m*, but a smaller number of observations, *N*. In addition, as can be seen, the RMSE for the projections behaves similarly for the Wiener and the Hammerstein structures. Besides, as the model order increases, the error in the projections increases. Notably, the minimum RMSE error in the projections is produced for the same values of *N* and *m* that minimize the RMSE for the model output, which indicates, as expected, that the performance of the decomposition algorithm is related to the performance of the model.

**Figure 5:**
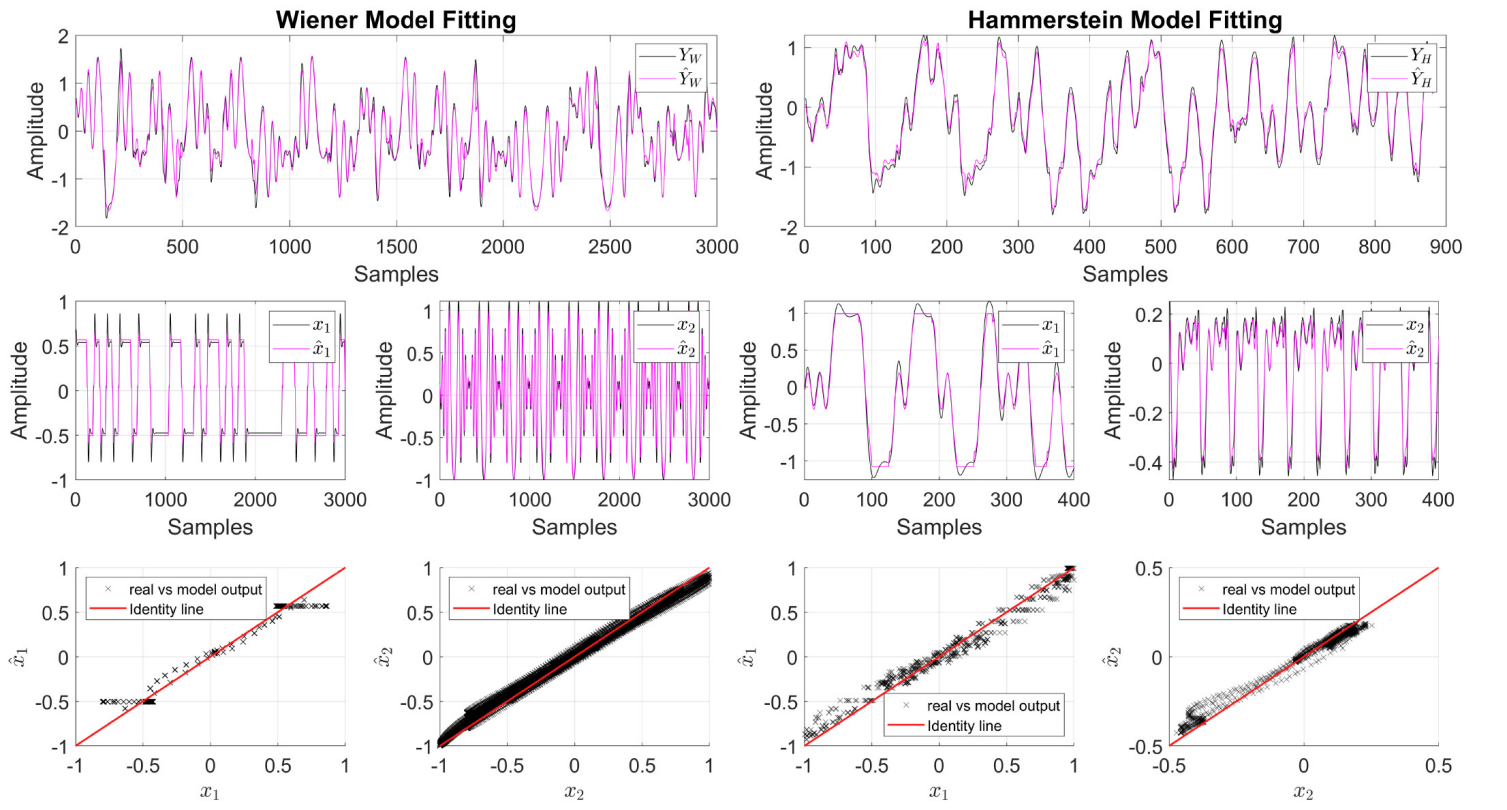
Results form NObSP applied to Wiener and Hammerstein block system structure. The first row displays the model fit in magenta and the real output in black. The second row presents the real nonlinear contribution of the input variables on the output and, in magenta, the estimated contributions using NObSP. The third row shows the calibration plots to analyze how well the algorithm predicts the projections. In some images, the x-axis limits are set for visualization effects; however, the behavior of the dynamic is similar in the entire interval.

Figure 5 presents the results for the model fit, as well as for the estimated contributions, ${\hat{x}}_{1}$ and ${\hat{x}}_{2}$, using test data. The first column shows the results for the Wiener structure, while the second column shows the results for the Hammerstein structure. The first row shows the estimated output of the model, the second row displays the estimated projections, and the third row presents the calibration plot to analyze the regression output. First, as can be seen in both cases, the model is able to predict the behavior of the output signal accurately. In the second row, it is shown that NObSP is able to accurately retrieve the signals **x**_1_ and **x**_2_. In the case of the Wiener structure, the dynamics of **x**_1_, generated using the PBRS signal, present some peaks that NObSP cannot reproduce. However, NObSP can estimate the nonlinear contributions for the general dynamics of the signal and the projections. The third row displays the calibration plots, showing that for ${\hat{x}}_{1}$ in the Wiener structure, the problem with the peaks causes horizontal lines that deviate from the identity line, which represents a model without errors. The calibration plot indicates a suitable dispersion around the identity line for the other projections. However, a small span error is observed, which is caused by errors due to scaling factors.

**Figure 6:**
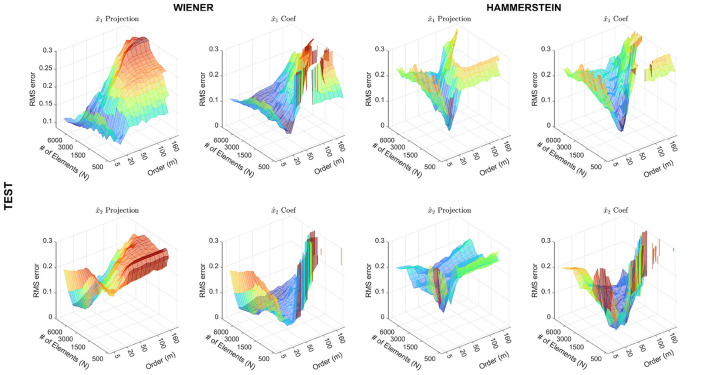
Partial (non)linear contributions of the input regressors on the output, for the test dataset, computed using the **α**_*l*_ coefficients.

Figure 6 presents the RMSE curves for a test set. Here, the behavior of the models has been evaluated for different values of *N* and *m*. The left columns display the results for the Wiener structure, while the right columns display the results for the Hammerstein structure. The first column shows the results using the projection matrices, and the second column shows the results from the out-of-sample extension. As expected, this figure displays a similar behavior of the RMSE for both the projections and the results obtained using the out-of-sample extension. It is important to highlight that the results for the out-of-sample extension present some peaks, which might be produced due to an ill-conditioned least-square problem, mainly caused by rank-deficient kernel matrices. This effect is more remarked for the Hammerstein system structure.

**Figure 7:**
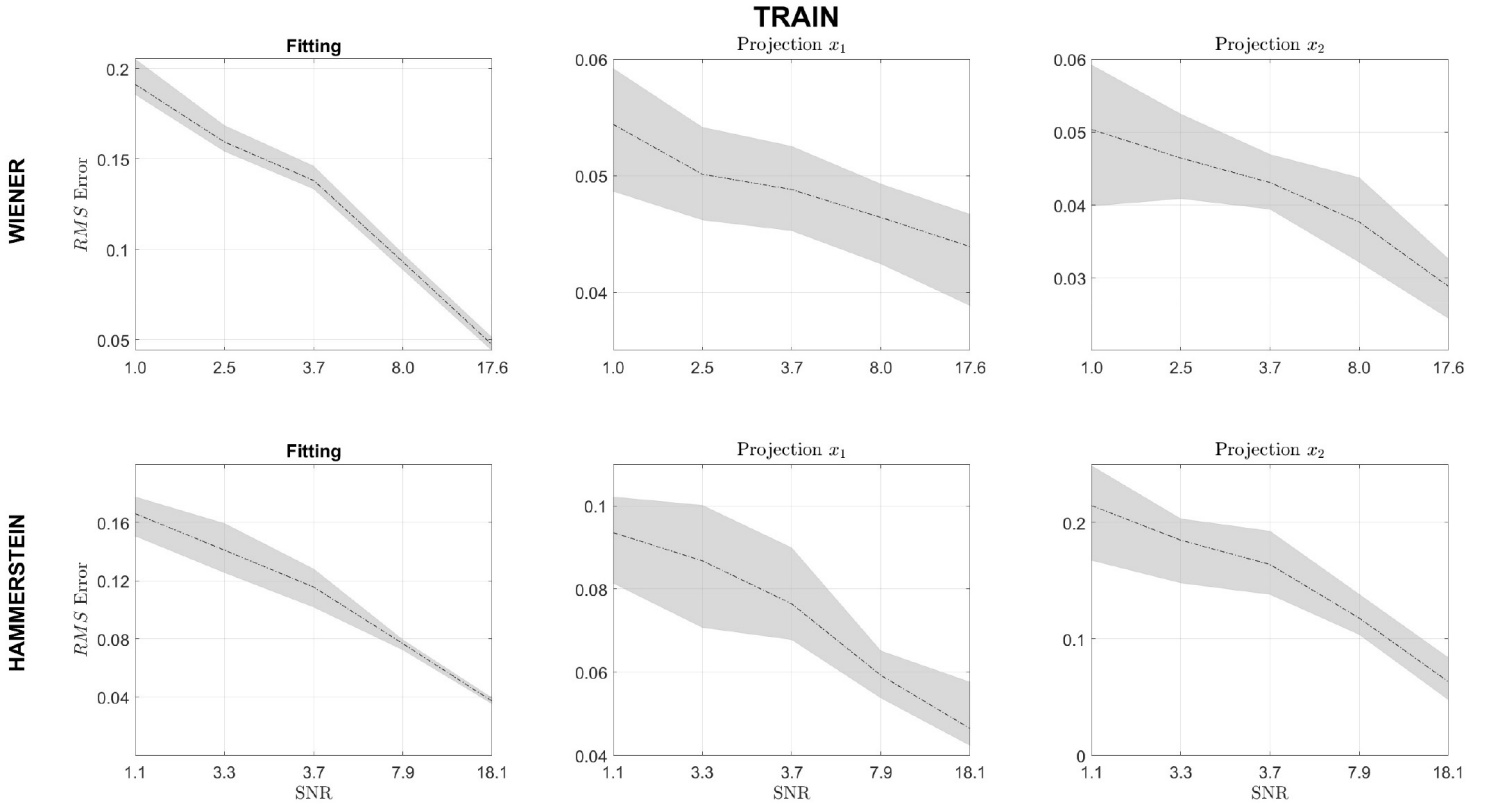
The solid black line represents the mean Normalized RMS error and the gray area the 95% confidence interval for fitting, projection ${\hat{x}}_{1}$ and projection ${\hat{x}}_{2}$ for different signal to noise ratio. The first row represents the Wiener structure, and the second one the Hammerstein structure.

Figure 7 displays the results for the changes in SNR. The first row presents the results for the Wiener block structure and the second for the Hammerstein block structure. The first column shows the results for the output fit, while the second and the third ones present the results for the projections ${\hat{x}}_{1}$ and ${\hat{x}}_{2}$. The figure shows that when SNR increases, the fit error decreases for both the Wiener and the Hammerstein systems, which is to be expected. Additionally, the projections, ${\hat{x}}_{1}$ and ${\hat{x}}_{2}$, for the Wiener system seem to be independent of the SNR within the range of variation. Similarly, the projections for the Hammerstein system seem to decrease; however, they exhibit a higher RMSE than the Wiener structure.

**Figure 8:**
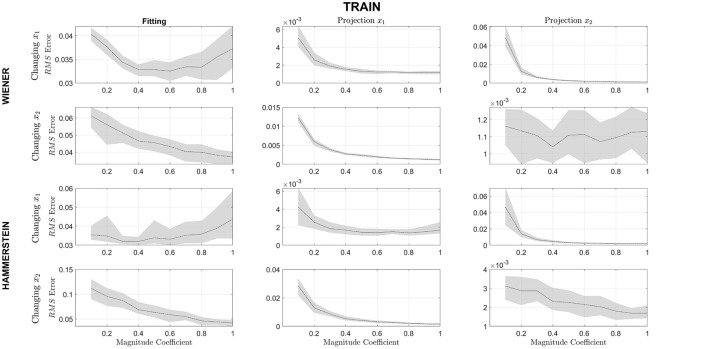
The solid black line represents the mean Normalized RMS error and the gray area the 95% confidence interval for Fitting, Projection ${\hat{x}}_{1}$ and Projection ${\hat{x}}_{2}$ for different magnitude coefficient. The upper half of the rows represent the Wiener structure, and the lower half represents the Hammerstein. In the first and third row, the changing signal is the *PRBS* signal (*u*_1_) and in the second and fourth row, the changing signal is the *sine* signal (*u*_2_).

Figure 8 presents the results for the changes in the relative amplitude between the signals **x**_1_ and **x**_2_. As can be seen, changing the relative amplitude of **x**_1_ in relation to **x**_2_, in both system structures, increases the error in the model fit, although not significantly. However, the error of the projections decreases. When changing the relative amplitude of **x**_2_ in relation to **x**_1_. It can be seen that the fit of the model improves when the amplitudes of the signal are equivalent. As in the previous case, the projection errors also decrease.

### 3.1 Computational performance validation

To quantitatively validate the practical impact of the computational cost reduction, a timing analysis was conducted. The experiment compares the execution time of two methods for decomposing the model output **ŷ**:

1. **NObSP:** The original approach, which requires the re-computation of the full projection matrices **P**_*l*/(*l*)_ (Equation 11) for the entire dataset. The complexity of this method is dominated by operations on the *N*×*N* kernel matrices, leading to 𝒪(*N*^3^) complexity.
2. **Out-of-sample extension:** The efficient method (Equation 16), which calculates the ${\hat{\alpha }}_{l}$ coefficients. The complexity of this step scales with 𝒪(*Nd*^2^), where *d* is the number of support vectors and, critically, *d*≪*N*.

The average execution time (over 15 simulations) was recorded for both methods across the full range of model orders (*m*) and sample sizes (*N*) used in the study. “Speedup Ratio” was then calculated as $\frac{\text{Time}\left(\text{Full NObSP}\right)}{\text{Time}\left(\text{OOS Extension}\right)}$ to quantify the relative performance gain.

**Figure 9:**
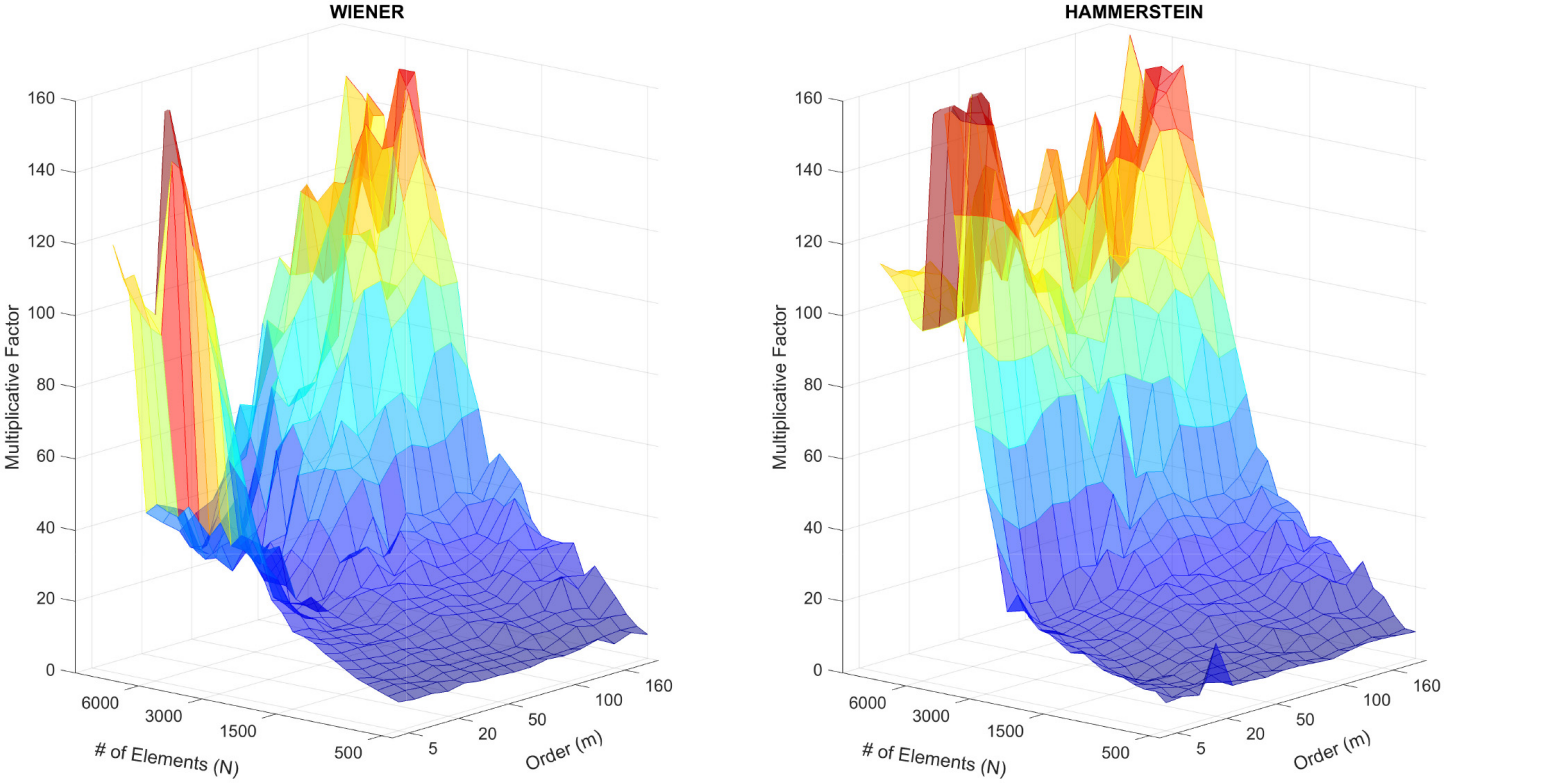
Computational speedup (ratio) of the Out-of-Sample (OOS) Extension compared to the NObSP method. The speedup, calculated as Time(NObSP)/Time(OOS Extension), is plotted as a function of the number of samples (*N*) and the model order (*m*) for both the **(left)** Wiener and **(right)** Hammerstein structures. The results demonstrate a significant performance gain that scales dramatically with *N*.

Figure 9 presents the results of this analysis for both the Wiener and Hammerstein system simulations. The data clearly shows a dramatic increase in the speedup ratio as the number of samples *N* grows, while the model order *m* has a comparatively minor impact. For the Wiener system, the OOS extension was found to be over 75 times faster, and for the Hammerstein system, over 147 times faster, at the largest sample size (*N* = 8, 000).

A non-monotonic behavior is also observed (i.e., the speedup ratio occasionally drops as *N* increases). This is likely attributable to the numerical properties of the kernel matrices being solved in NObSP method (Equation 16). The efficiency of the underlying numerical solver used to compute the pseudoinverse can vary depending on the specific characteristics of the matrix at different scales (e.g., its condition number), causing these local variations in computational time.

Despite this numerical variance, the overall behavior is consistent with the theoretical complexity. The 𝒪(*N*^3^) complexity of the Full NObSP method causes its execution time to grow cubically, becoming computationally prohibitive for large datasets. Conversely, the OOS extension's cost scales much more favorably, as it avoids operations on the large *N*×*N* matrices. This experiment provides strong quantitative validation—demonstrating a speedup of more than two orders of magnitude—that the OOS extension is not just theoretically, but also practically, superior for large-scale system identification problems.

## 4 Discussion

This paper presents an extension of NObSP for the decomposition of the output of a nonlinear dynamical system. The extension allows the decomposition of a model, initially identified as a nonlinear mean average system using SVM, into additive components where each one represents the nonlinear contribution of each input signal on the dynamics of the output. In the literature, some methodologies exist that allow retrieving the functional relationship between inputs-outputs in a black-box model. However, these methods require to define a priori the relevant effects of interest for the designer, which can bias the identified model, e.g., functional ANOVA models (Abramovich and Angelini, 2006). Other *post hoc* methods can retrieve the functional relation for static models, such as the Partial Dependence plot (PDP) (Burkart and Huber, 2021). In addition, Volterra series can be used for dynamic system identification. However, a review of the literature suggests that this method does not allow to estimate the partial nonlinear dynamic contribution of each independent input (Cheng et al., 2017; Dalla Libera et al., 2021). Likewise, in (Pei et al. 2022), the authors proposed a framework to replicate traditional methods for structural health monitoring using sigmoidal neural networks, which improves the interpretability of the resulting model. (Pei et al. 2022), used domain knowledge to identify the dominant features and approximate their contributions using sigmoidal neural networks, thereby fitting the specified function approximation using a linear combination of these learned features. Even though these sigmoidal functions are generic, this method is specific for NN and imposes a specific model architecture by the association of some types of nonlinearities, which can incorporate domain knowledge. Additionally, in the context of optimization, this training method produces a local search. In contrast, NObSP is a more general framework since it allows the retrieval of the nonlinear contribution of the inputs without additional processing from an already trained black box model. In this sense, the global search developed by the minimization of the approximation error in the training process is not biased by NObSP methodology, and the functional relation between the input regressors and the output is not restricted by any condition. These characteristics allow the use of NObSP in different domains without the necessity of domain knowledge. NObSP was initially developed for LS-SVM static regression models by (Caicedo et al. 2019). This study shows that NObSP is able to retrieve the partial nonlinear dynamic contribution of each input variable on the output for Wiener and Hammerstein block-system structures. In Figure 4, it is observed that the goodness of the model fit directly impacts the performance of NObSP. Therefore, in order to obtain an adequate decomposition, it is crucial first to have a model that fits the data satisfactorily.

In general, SVM performs a nonlinear transformation on the input data and maps it to a Hilbert space, facilitating the solution of the regression problem on the transformed space since, in this space, the model is considered linear. For this reason, SVM is only able to identify functions that lie on a Hilbert space. Interestingly, the kernel matrix represents a low-rank approximation of the hyperplane where the transformed data lies, which in turn means a low-rank approximation of the manifold of the identified model. Since the number of observations, *N*, determines the size of the kernel matrices, it impacts the projections due to the fact that the maximum rank for the kernel matrix is *N*. More specifically, the size of the kernel determines the number of vectors that represent the column space of the hyperplane in the Hilbert space. Considering this fact, Wiener structures seem to generate subspaces of larger dimensions, while the Hammerstein model needs fewer basis vectors to span the subspace of the nonlinear transformations. This issue might indicate that the manifold where the input and output observations lie might be more complex for the Wiener structure than for the Hammerstein structure.

Another approach to the out-of-sample extension is to consider that **ŷ**_*l*_ = **P**_*l*/(*l*)_**ŷ**, then **ŷ**_*l*_ = **P**_*l*/(*l*)_**Ω**_*C*_**α** = **Ω**_*C*_**α**_*l*_, where $\alpha_{l}\in {\mathbb{R}}^{r}$. And solving using least squares, it is obtained ${\hat{\alpha }}_{l}={\left({\text{Ω}}_{C}^{\text{T}}{\text{Ω}}_{C}\right)}^{†}{\text{Ω}}_{C}^{\text{T}}{ŷ}_{l}$. The decomposition of the output model ${ŷ}_{x_{j}}=\text{K}\left(\text{x},{\text{x}}_{\text{SV}}\right){\hat{\alpha }}_{x_{j}}$ retrieved by the ${\hat{\alpha }}_{x_{j}}$ coefficients just need one evaluation of the kernel but the output is noisier than that given by the projections. This issue may be caused by the fact that there is an overlap of the subspaces, i.e., ${⋂}_{k=1}^{r}𝒞\left({\text{Ω}}_{k}\right)\neq \emptyset$, where 𝒞(**Ω**_*j*_) = *Span*(**Ω**_*j*_). The subspaces for each input variable are not disjointed, probably due to the low-rank approximation caused by the number of support vectors and the number of data samples. Nevertheless, the decomposition of the output model using ${\hat{\alpha }}_{x_{j}}$ coefficients obtained by solving weighted least squares, i.e., $y_{x_{j}}=\text{K}\left({\text{x}}_{j},{\text{x}}_{\text{SV}}\right){\hat{\alpha }}_{x_{j}}$ produces the same results as the projection matrices since **ŷ**_*x*_*j*__⊂𝒞(**Ω**_*j*_). However, to compute the contribution of each input regressor, the kernel function needs to be evaluated.

In addition, concerning the out-of-sample extension, it was shown that the $\hat{\alpha }$ coefficients can capture the dynamics of the system, largely reducing NObSP computational time and complexity. However, in some cases, ill-conditioned matrices can produce inflated coefficients, which negatively impacts the performance of the decomposition. Here, it is important to take into consideration several aspects. First, SVM is able to reproduce functions that lie in a Hilbert space. Regarding the application of the methodology to more complex systems beyond the tested Wiener and Hammerstein structures, the theoretical limits of the SVM model itself must be considered. SVMs, and specifically the LS-SVM framework used in this work, are established as universal approximators for functions that reside within a Reproducing Kernel Hilbert Space (RKHS) (Suykens J. A. K. et al., 2002; Vapnik, 1999).

These spaces are known to contain functions that are smooth and continuous. The NObSP algorithm, being a *post-hoc* geometric methodology, is designed to decompose the function *already learned* by the SVM. Therefore, it is intuited that NObSP can successfully retrieve the partial components of any system that the underlying SVM can accurately model. As long as the system's nonlinear dynamics are not so irregular as to fall outside the RKHS (e.g., highly discontinuous functions) that the SVM is capable of approximating, the decomposition is expected to be valid.

A formal mathematical proof defining the precise functional boundaries (e.g., which specific compositions of *f*(·) and *G*(*q*)) are identifiable by an SVM and, subsequently, decomposable by NObSP remains a complex and open question that is outside the scope of this paper. Furthermore, the practical success of this decomposition relies on the numerical stability of the kernel matrices (Equation 11), which must be well-conditioned (Caicedo et al., 2019).

Considering the changes in the SNR of the output signal, it can be seen in Figure 6 that the fit of the model and the estimated projections ${\hat{x}}_{1}$ and ${\hat{x}}_{2}$ improves at the SNR increase. This behavior is expected since the quality of the projections depends directly on the quality of the model fit. For this reason, before applying the decomposition, it is important to guarantee that the model fit is accurate. If this condition is not fulfilled, then NObSP will not perform properly. In such cases, the estimated manifold where the input and output observation lies might be under-fitted or over-fitted. The effect of each one of those phenomena on the projections is yet to be studied.

Concerning the changes in the relative magnitudes of the estimated projections, it is shown in Figure 8 that when the signals are comparable in magnitude, the normalized RMSE value is lower than when there is a significant relative difference in magnitude. Interestingly enough, increasing the magnitude of the component **x**_1_ reduces the RMSE for the fitting in the model output. When the PBRS signal is analyzed, it can be found that it does not belong to a Hilbert space but in a Bounded Variation space since its second derivative is discontinuous (Parhi and Nowak, 2021). Therefore, by increasing the relevance of the discontinuous components on the output, the output signal diverges more and more from a Hilbert space. In Figure 6, it can be seen that for the Wiener structure, the borders of ${\hat{x}}_{1}$ are not well captured by NObSP, which is where the second derivative of the function is discontinuous.

To finalize, it is important to note that the main objective of this paper is not to identify whether the system contains a Wiener or a Hammerstein structure nor to propose a new identification algorithm but to provide an algorithm to decompose the output of an already identified system into independent components related to each input variable. In this sense and outside of the scope discussed in (Rudin 2019), the methodology proposed in this manuscript begins with an accurate black-box model that could predict the behavior of the system. Based on this model and using a geometric approach, the interpretation algorithm projects the influence of each input regressor over the other regressors using the kernel matrix of the model, obtaining the marginal functional relation between the input regressors and the output. Once these signals are obtained, in the literature, there exist several methods that are able to identify each component of a Wiener or a Hammerstein structure (Castro-Garcia and Suykens, 2016; Bottegal et al., 2018; Bottegai et al., 2017; Falck et al., 2009). In addition, some open questions require further studies, such as: What type of composition of input signals, nonlinear functions, and linear transformations can be identified accurately using SVM? Can NObSP be applied to other nonlinear identification methods? Can NObSP allow a suitable retrieval of the second-order interaction between inputs?

## 5 Conclusion

This research demonstrates that Non-linear Oblique Subspace Projections (NObSP) are a viable and effective method for retrieving interpretability from black-box Support Vector Machine (SVM) models used in dynamic system identification. This work showed that a rigorous geometric decomposition, specifically one that handles correlated regressors, can successfully retrieve the blended dynamics of a nonlinear system. This moves beyond simple feature attribution by reconstructing the *full, non-parametric contribution* of each input regressor. As the simulation results in the previous section confirmed, NObSP effectively decomposed the identified SVM model into its constituent Wiener and Hammerstein sub-systems, accurately retrieving the partial dynamics. The method's robustness was also validated against significant signal noise, showing that decomposition quality improves as the Signal-to-Noise Ratio (SNR) increases.

The research findings also provide insight into the modeling of nonlinear dynamics. The analysis of the training requirements showed that the Wiener structure requires a larger number of training samples (*N*) than the Hammerstein structure for an accurate fit. This suggests a higher functional complexity in the identified manifold, characterized by the presence of more abrupt signal changes and discontinuities. These discontinuities require a richer, higher-dimensional basis (i.e., more support vectors) for the SVM to model accurately. On the practical side, this work validated an efficient out-of-sample extension. The computational analysis demonstrated a dramatic reduction in execution time, confirming that the 𝒪(*Nd*^2^) method is scalable and practically superior in executipon time, while producing comparable decomposition results to the 𝒪(*N*^3^) approach for large datasets.

Future research should focus on extending this geometric framework to other black-box models. Extending NObSP to neural networks, for example, is a significant challenge, as it would require developing a new mathematical formulation to handle the lack of an explicit kernel structure and to manage the high-dimensional, coupled nonlinearities introduced by activation functions. This validation of NObSP on sequential, dynamic data also suggests a promising direction for future work. It demonstrates that a rigorous geometric decomposition is a viable methodology for interpreting sequential data, offering a path toward true functional decomposition in other complex sequential model architectures, such as Transformers or Long Short-Term Memory (LSTMs).

Finally, while this work validated the methodology robustly using established simulation benchmarks (Wiener and Hammerstein), a crucial next step is the application of this framework to real-world experimental data. This will be essential to test the method's performance against non-ideal conditions, such as unmodeled cross-couplings and non-Gaussian noise, which are common in physical and industrial processes.

## Data Availability

The datasets presented in this study can be found in online repositories. The names of the repository/repositories and accession number(s) can be found in the article/supplementary material.

## Footnotes

1 The code used for the simulations, for reproducibility of the results, can be found in the following repository: https://github.com/JoeCode91/EAAI_SystemDynamicIdenification_Interpretability.

## References

[B1] Abramovich F. Angelini C. (2006). Testing in mixed-effects FANOVA models. J. Stat. Plan. Inference 136, 4326–4348. doi: 10.1016/j.jspi.2005.06.002

[B2] Agarwal R. Melnick L. Frosst N. Zhang X. Lengerich B. Caruana R. . (2021). “Neural additive models: interpretable machine learning with neural nets,” in Advances in Neural Information Processing Systems, 4699–4711.

[B3] Angelini M. Blasilli G. Lenti S. Santucci G. (2023). A visual analytics conceptual framework for explorable and steerable partial dependence analysis. IEEE Trans. Vis. Comput. Graph. 30, 4497–4513. doi: 10.1109/TVCG.2023.3263739 37027262

[B4] Aquino G. Costa M. Filho C. (2022). Explaining one-dimensional convolutional models in human activity recognition and biometric identification tasks. Sensors 22:5644. doi: 10.3390/s22155644 35957201 PMC9371158

[B5] Barredo Arrieta A. Díaz-Rodríguez N. Del Ser J. Bennetot A. Tabik S. Barbado A. . (2020). Explainable Artificial Intelligence (XAI): concepts, taxonomies, opportunities and challenges toward responsible AI. Inf. Fusion 58, 82–115. doi: 10.1016/j.inffus.2019.12.012

[B6] Bottegai G. Castro-Garcia R. Suykens J. A. (2017). “On the identification of wiener systems with polynomial nonlinearity,” in 2017 IEEE 56th Annual Conference on Decision and Control (CDC) (IEEE), 6475–6480. doi: 10.1109/CDC.2017.8264635

[B7] Bottegal G. Castro-Garcia R. Suykens J. A. (2018). A two-experiment approach to wiener system identification. Automatica 93, 282–289. doi: 10.1016/j.automatica.2018.03.069

[B8] Bring J. (1996). A geometric approach to compare variables in a regression model. Am. Stat. 50, 57–62. doi: 10.1080/00031305.1996.10473543

[B9] Burkart N. Huber M. F. (2021). A survey on the explainability of supervised machine learning. J. Artif. Intell. Res. 70, 245–317. doi: 10.1613/jair.1.12228

[B10] Caicedo A. Varon C. Huffel S. V. Suykens J. A. (2019). Functional form estimation using oblique projection matrices for ls-SVM regression models. PLoS ONE 14, 1–21. doi: 10.1371/journal.pone.0217967 31173619 PMC6555528

[B11] Candon M. Esposito M. Fayek H. Levinski O. Koschel S. Joseph N. . (2022). Advanced multi-input system identification for next generation aircraft loads monitoring using linear regression, neural networks and deep learning. Mech. Syst. Signal Process. 171:108809. doi: 10.1016/j.ymssp.2022.108809

[B12] Castro-Garcia R. Suykens J. A. (2016). “Wiener system identification using best linear approximation within the ls-svm framework,” in 2016 IEEE Latin American Conference on Computational Intelligence (LA-CCI) (IEEE), 1–6. doi: 10.1109/LA-CCI.2016.7885698

[B13] Cheng C. Peng Z. Zhang W. Meng G. (2017). Volterra-series-based nonlinear system modeling and its engineering applications: a state-of-the-art review. Mech. Syst. Signal Process. 87, 340–364. doi: 10.1016/j.ymssp.2016.10.029

[B14] Dalla Libera A. Carli R. Pillonetto G. (2021). Kernel-based methods for volterra series identification. Automatica 129:109686. doi: 10.1016/j.automatica.2021.109686

[B15] DeVore R. Petrova G. Wojtaszczyk P. (2011). Approximation of functions of few variables in high dimensions. Constr. Approx. 33, 125–143. doi: 10.1007/s00365-010-9105-8

[B16] Espinoza M. Suykens J. A. De Moor B. (2005). Kernel based partially linear models and nonlinear identification. IEEE Trans. Automat. Contr. 50, 1602–1606. doi: 10.1109/TAC.2005.856656

[B17] Falck T. Pelckmans K. Suykens J. A. De Moor B. (2009). Identification of wiener-hammerstein systems using ls-svms. IFAC Proc. 42, 820–825. doi: 10.3182/20090706-3-FR-2004.00136

[B18] Forgione M. Muni A. Piga D. Gallieri M. (2023). On the adaptation of recurrent neural networks for system identification. Automatica 155:111092. doi: 10.1016/j.automatica.2023.111092

[B19] Goethals I. Pelckmans K. Hoegaerts L. Suykens J. De Moor B. (2005). “Subspace intersection identification of hammerstein-wiener systems,” in Proceedings of the 44th IEEE Conference on Decision and Control (IEEE), 7108–7113. doi: 10.1109/CDC.2005.1583307

[B20] Gonzalez-Olvera M. A. Tang Y. (2010). Black-box identification of a class of nonlinear systems by a recurrent neurofuzzy network. IEEE Trans. Neural Netw. 21, 672–679. doi: 10.1109/TNN.2010.2041068 20172820

[B21] Harrell F. E. (2001). Regression Modeling Strategies: With Applications to Linear Models, Logistic Regression, and Survival Analysis. Cham: Springer. doi: 10.1007/978-1-4757-3462-1

[B22] Jutte A. Ahmed F. Linssen J. Keulen M. V. (2025). C-shap for time series: an approach to high-level temporal explanations. ArXiv, abs/2504.11159.

[B23] Li F. Liang M. He N. Cao Q. (2023a). Separation identification approach for the Hammerstein-Wiener nonlinear systems with process noise using correlation analysis. Int. J. Robust Nonl. Control 33, 8105–8123. doi: 10.1002/rnc.6731

[B24] Li F. Liang M. Luo Y. (2023b). Correlation analysis-based parameter learning of Hammerstein nonlinear systems with output noise. Eur. J. Control 72:100819. doi: 10.1016/j.ejcon.2023.100819

[B25] Li F. Qian S. He N. Li B. (2024). Estimation of wiener nonlinear systems with measurement noises utilizing correlation analysis and Kalman filter. Int. J. Robust Nonl. Control 34, 4706–4718. doi: 10.1002/rnc.7224

[B26] Li F. Zheng T. He N. Cao Q. (2022). Data-driven hybrid neural fuzzy network and ARX modeling approach to practical industrial process identification. IEEE/CAA J. Autom. Sinica 9, 1702–1705. doi: 10.1109/JAS.2022.105821

[B27] Li F. Zhu X. Cao Q. (2023c). Parameter learning for the nonlinear system described by a class of Hammerstein models. Circ. Syst. Signal Proc. 42, 2635–2653. doi: 10.1007/s00034-022-02240-y

[B28] Li J. Zong T. Lu G. (2022). Parameter identification of Hammerstein–Wiener nonlinear systems with unknown time delay based on the linear variable weight particle swarm optimization. ISA Trans. 120, 89–98. doi: 10.1016/j.isatra.2021.03.021 33814264

[B29] Ljung L. Andersson C. Tiels K. Schön T. B. (2020). Deep learning and system identification. IFAC-PapersOnLine 53, 1175–1181. doi: 10.1016/j.ifacol.2020.12.1329

[B30] Luckey D. Fritz H. Legatiuk D. Peralta Abadía J. J. Walther C. Smarsly K. (2022). Explainable Artificial Intelligence to Advance Structural Health Monitoring. Cham: Springer International Publishing, 331–346. doi: 10.1007/978-3-030-81716-9_16

[B31] Lundberg S. M. Lee S.-I. (2017). “A unified approach to interpreting model predictions,” in Proceedings of the 31st International Conference on Neural Information Processing Systems, NIPS'17 (Red Hook, NY, USA: Curran Associates Inc.), 4768–4777.

[B32] Parhi R. Nowak R. D. (2021). Banach space representer theorems for neural networks and ridge splines. J. Mach. Learn. Res. 22, 1960–1999. doi: 10.48550/arXiv.2006.05626

[B33] Pei J.-S. Hougen D. F. Kanneganti S. T. Wright J. P. Mai E. C. Smyth A. W. . (2022). Interpretable Machine Learning for Function Approximation in Structural Health Monitoring. Cham: Springer International Publishing, 369–388. doi: 10.1007/978-3-030-81716-9_18

[B34] Pena-Campos J. Patino D. Ocampo-Martinez C. Caicedo A. (2023). “Out-of-sample extension of kernel-based interpretation models for SVM regression using oblique subspace projections,” in World Congress of the International Federation of Automatic Control. Electronic Proceedings of the IFAC World Congress 2023 (Yokohama, Japan).

[B35] Ravikumar P. Lafferty J. Liu H. Wasserman L. (2009). Sparse additive models. J. R. Statist. Soc. Series B 71, 1009–1030. doi: 10.1111/j.1467-9868.2009.00718.x

[B36] Resendiz-Trejo J. A. Yu W. Li X. (2006). “Support vector machine for nonlinear system on-line identification,” in 2006 3rd International Conference on Electrical and Electronics Engineering (IEEE), 1–4. doi: 10.1109/ICEEE.2006.251894

[B37] Ribeiro M. T. Singh S. Guestrin C. (2016). “Why should i trust you?”: explaining the predictions of any classifier,” in *Proceedings of the 22nd ACM SIGKDD International Conference on Knowledge Discovery and Data Mining, KDD '16* (New York, NY, USA: ACM), 1135–1144. doi: 10.1145/2939672.2939778

[B38] Rojat T. Puget R. Filliat D. Ser J. D. Gelin R. Díaz-Rodríguez N. (2021). Explainable artificial intelligence (xAI) on timeseries data: a survey. arXiv preprint arXiv:2104.00950.

[B39] Rudin C. (2019). Stop explaining black box machine learning models for high stakes decisions and use interpretable models instead. Nat. Mach. Intell. 1, 206–215. doi: 10.1038/s42256-019-0048-x 35603010 PMC9122117

[B40] Selvaraju R. R. Das A. Vedantam R. Cogswell M. Parikh D. Batra D. (2016). Grad-cam: visual explanations from deep networks via gradient-based localization. Int. J. Comput. Vis. 128, 336–359. doi: 10.1007/s11263-019-01228-7

[B41] Sen D. Deora B. S. Vaishnav A. (2025). Explainable deep learning for time series analysis: integrating SHAP and LIME in LSTM-based models. J. Inf. Syst. Eng. Manag. 10, 412–423. doi: 10.52783/jisem.v10i16s.2627

[B42] Shi H. Yang N. Yang X. Tang H. (2023). Clarifying relationship between pm2.5 concentrations and spatiotemporal predictors using multi-way partial dependence plots. Remote. Sens. 15:358. doi: 10.3390/rs15020358

[B43] Suykens J. De Brabanter J. Lukas L. Vandewalle J. (2002). Weighted least squares support vector machines: robustness and sparse approximation. Neurocomputing 48, 85–105. doi: 10.1016/S0925-2312(01)00644-0

[B44] Suykens J. A. K. Van Gestel T. De Brabanter J. De Moor B. Vandewalle J. (2002). Least Squares Support Vector Machines. Singapore: World Scientific. doi: 10.1142/5089

[B45] Theissler A. Spinnato F. Schlegel U. Guidotti R. (2022). Explainable ai for time series classification: a review, taxonomy and research directions. IEEE Access 10, 100700–100724. doi: 10.1109/ACCESS.2022.3207765

[B46] Van Belle V. Lisboa P. (2014). White box radial basis function classifiers with component selection for clinical prediction models. Artif. Intell. Med. 60, 53–64. doi: 10.1016/j.artmed.2013.10.001 24262978

[B47] Vapnik V. N. (1999). An overview of statistical learning theory. IEEE Trans. Neural Netw. 10, 988–999. doi: 10.1109/72.788640 18252602

[B48] Yazdani A. Lu L. Raissi M. Karniadakis G. E. (2020). Systems biology informed deep learning for inferring parameters and hidden dynamics. PLoS Comput. Biol. 16, 1–20. doi: 10.1371/journal.pcbi.1007575 33206658 PMC7710119

[B49] Yeh I.-C. (1998). Modeling of strength of high-performance concrete using artificial neural networks. Cement Concr. Res. 28, 1797–1808. doi: 10.1016/S0008-8846(98)00165-3

[B50] Zong T. Li J. Lu G. (2021). Auxiliary model-based multi-innovation PSO identification for Wiener–Hammerstein systems with scarce measurements. Eng. Appl. Artif. Intell. 106:104470. doi: 10.1016/j.engappai.2021.104470

