# Correction: Retrieving interpretability to support vector machine regression models in dynamic system identification

**DOI:** 10.3389/frai.2026.1857609

**Published:** 2026-04-29

**Authors:** Johan Pena-Campos, Diego Patino, Carlos Ocampo-Martinez, Julio C. Ramos-Fernández, Margot Salas-Brown, Alexander Caicedo

**Affiliations:** 1 Department of Electronic Engineering, Pontificia Universidad Javeriana, Bogota, Colombia; 2 Automatic Control Department (ESAII), Universitat Politecnica de Catalunya, Barcelona, Spain; 3 Faculty of Mathematical and Natural Sciences, Universidad Distrital Francisco José de Caldas, Bogotá, Colombia; 4 School of Exact Sciences and Engineering, Universidad Sergio Arboleda, Bogotá, Colombia; 5 Ressolve SAS, Medelln, Colombia

**Keywords:** interpretability, oblique projections, support vector machine, Hammerstein-Wiener models, dynamic systems, system identification

In the published article, the order of the affiliations was incorrect. The affiliations were originally listed as:

^1^ Automatic Control Department (ESAII), Universitat Politecnica de Catalunya, Barcelona, Spain

^2^ Department of Electronic Engineering, Pontificia Universidad Javeriana, Bogota, Colombia

The correct affiliation order is:

^1^ Department of Electronic Engineering, Pontificia Universidad Javeriana, Bogota, Colombia

^2^ Automatic Control Department (ESAII), Universitat Politecnica de Catalunya, Barcelona, Spain

Accordingly, the author affiliation numbers have been updated to reflect the corrected affiliation order. Affiliations 3, 4, and 5 remain unchanged.

The corresponding authors were erroneously listed as:

johan.sebastian.pena@upc.edu; pena.johan@javeriana.edu.co.

The correct corresponding author list is: pena.johan@javeriana.edu.co; johan.sebastian.pena@upc.edu

The original version of this article has been updated.

